# Neotropical ostracode oxygen and carbon isotope signatures: implications for calcification conditions

**DOI:** 10.1007/s10533-022-00917-9

**Published:** 2022-03-29

**Authors:** Claudia Wrozyna, Juliane Meyer, Martin Dietzel, Werner E. Piller

**Affiliations:** 1 Institute of Geography and Geology, University of Greifswald, Friedrich-Ludwig-Jahn-Str. 17a, 17489 Greifswald, Germany; 2 NAWI Graz Geocenter, Institute of Earth Sciences, University of Graz, Heinrichstraße 26, 8010 Graz, Austria; 3 Institute of Applied Geosciences, Graz University of Technology, Rechbauerstraße 12, 8010 Graz, Austria

**Keywords:** Lakes, Hydrochemistry, Oxygen isotopes, Carbon isotopes, Neotropics, Authigenic carbonates, Ostracodes, Paleoclimate

## Abstract

Calcitic valves of non-marine ostracodes are important geochemical archives. Investigations of the relationship between the ranges of oxygen and carbon isotope values of modern ostracode populations and their host water provide important information on local or regional conditions and influences. Here we present the first δ^18^O_ostracode_ and δ^13^C of the freshwater ostracode species *Cytheridella ilosvayi* along with the isotopic composition of the waters in which the ostracodes calcified, δD_water_, δ^18^O_water_, δ^13^C_DIC_ values—covering a large geographical range (Florida to Brazil). With this data we extended a newly developed approach based on the estimation of δ^18^O values of monthly equilibrium calcites as references for the interpretation of δ^18^O_ostracode_ values. The expected apparent oxygen isotope fractionation between CaCO_3_ and H_2_O is correlated with temperature with smaller values occurring at higher temperatures as valid at isotope equilibrium (δ^18^O_calcite___eq_). Uncertainties about the expected equilibrium calcites derive from incomplete knowledge of high-frequency variations of the water bodies caused by interplay of mixing, evaporation, and temperature. Coincidence between δ^18^O_ostracode_ and δ^18^O_calcite___eq_ is restricted to few months indicating a seasonal calcification of *Cytheridella*. There is a characteristic pattern in its difference between mean δ^18^O_ostracode_ and δ^18^O_calcite___eq_ which implies that *Cytheridella* provides a synchronous life cycle in its geographical range with two calcification periods in spring (May, June) and autumn (October). This ubiquitous life cycle of *Cytheridella* in the entire study area is considered to be phylogenetically inherited. It might have originally been adapted to environmental conditions but has been conserved during the migration and radiation of the group over the Neotropical realm.

**Supplementary Information:**

The online version contains supplementary material available at 10.1007/s10533-022-00917-9.

## Introduction

Oxygen and carbon isotope measurements of ostracode valves from lake sediments are used extensively for the reconstruction of paleoclimatic conditions in continental settings. Uncertainties in interpretation of stable isotope records of ostracodes derive from incomplete knowledge on biomineralization processes, lake hydrochemistry, and ostracode autecology (Decrouy et al. 2011; Escobar et al. 2010). Ostracodes grow by molting up to nine growth stages (instars) (Aguilar-Alberola and Mesquita-Joanes 2013) which occur in a very short time of hours to few days (Turpen and Angell 1971) providing hydrochemical `snapshots´ of lake water at time of valve calcification. Therefore, in non-marine settings ostracodes are the only organism group producing authigenic skeletal carbonates that integrate short-term meteorological fluctuations such as seasonal changes (Escobar et al. 2010) or even paleo-storms (Lane et al. 2017). Additionally, ostracode species provide different life histories with either eurychronic forms in which adults calcify their valves throughout the year or stenochronic forms in which calcification is seasonally restricted (Meisch 2000).

As a result, ostracod assemblages within a stratigraphic layer are composed of multiple generations of valves that formed at different times, and the δ^18^O and δ^13^C of multiple valves reflects the average conditions over which the individuals lived (Dixit et al. 2015). Within recent years, detection limits of mass spectrometers continuously decreased facilitating single valve measurements (e.g., Escobar et al. 2010; Meyer et al. 2017a), which enables to receive a high-resolution climatic and/or environmental record.

Disentangling the individual contributions of the meteorological and climatical variability to the inter-valve isotopical variability within a sample represents the important key to generally improve the significance of ostracode stable isotope records. This represents a prerequisite for their use for reconstructions of climatic and environmental events. This requires precise knowledge on the species-specific life histories including the time of valve calcification and in-depth knowledge of hydrochemical characteristics. However, even by repeated samplings as usually done (e.g., Heip 1976; Schweitzer and Lohmann 1990; Decrouy and Vennemann 2014), it remains almost impossible to determine the actual time of calcification in the field. Additionally, hydrochemical and isotopic characteristics of non-marine water bodies often vary strongly on short time scales. Thus, it can be reasonably assumed that composition of the solution at time of sampling might not correspond to the solution in which (all) ostracode valves were formed.

In a former field study, Meyer et al. (2017b) successfully developed a new approach in which the oxygen isotope compositions of water and precipitation were used along with temperature to predict monthly oxygen isotope composition of calcites precipitated close to oxygen isotopic equilibrium. These were compared to δ^18^O_ostracode_ values of *Cytheridella ilosvayi*—a widespread freshwater ostracode—in order to determine regional and local influences on ostracode isotope composition and eventually identify calcification periods.

Generally, studies of modern ostracode stable isotope compositions and related solutions are restricted to small-scaled study areas or even individual water bodies (e.g., Decrouy et al. 2011; Marco-Barba et al. 2012; Pérez et al. 2013) based on time and analytical effort needed for sample processing. The resulting data sets and inferences on influences on stable oxygen and carbon isotopes are therefore quite specific. In the present study a highly geographically extended dataset of stable isotope composition of *C. ilosvayi* (δ^18^O_ostracode_, δ^13^C_ostracode_) and lake water (δD_water_, δ^18^O_water_, δ^13^C_DIC_) as well as water chemistry and temperature are used for the first time to verify and extend the above-described approach to the herein measured and collected widespread Neotropical ostracode data set.

Main tasks of the present study are (1) characterization of the relationships between mean isotopic signatures of ostracodes and lake water; (2) identification of possible regional-specific differences on the accuracy of monthly expected equilibrium calcites; and (3) inferences about calcification periods of *C. ilosvayi* within its geographical range. Our approach provides important data which are mandatory to utilize *C. ilosvayi* as proxy in paleolimnological and -climatological studies.

## Study areas

**Fig. 1:**
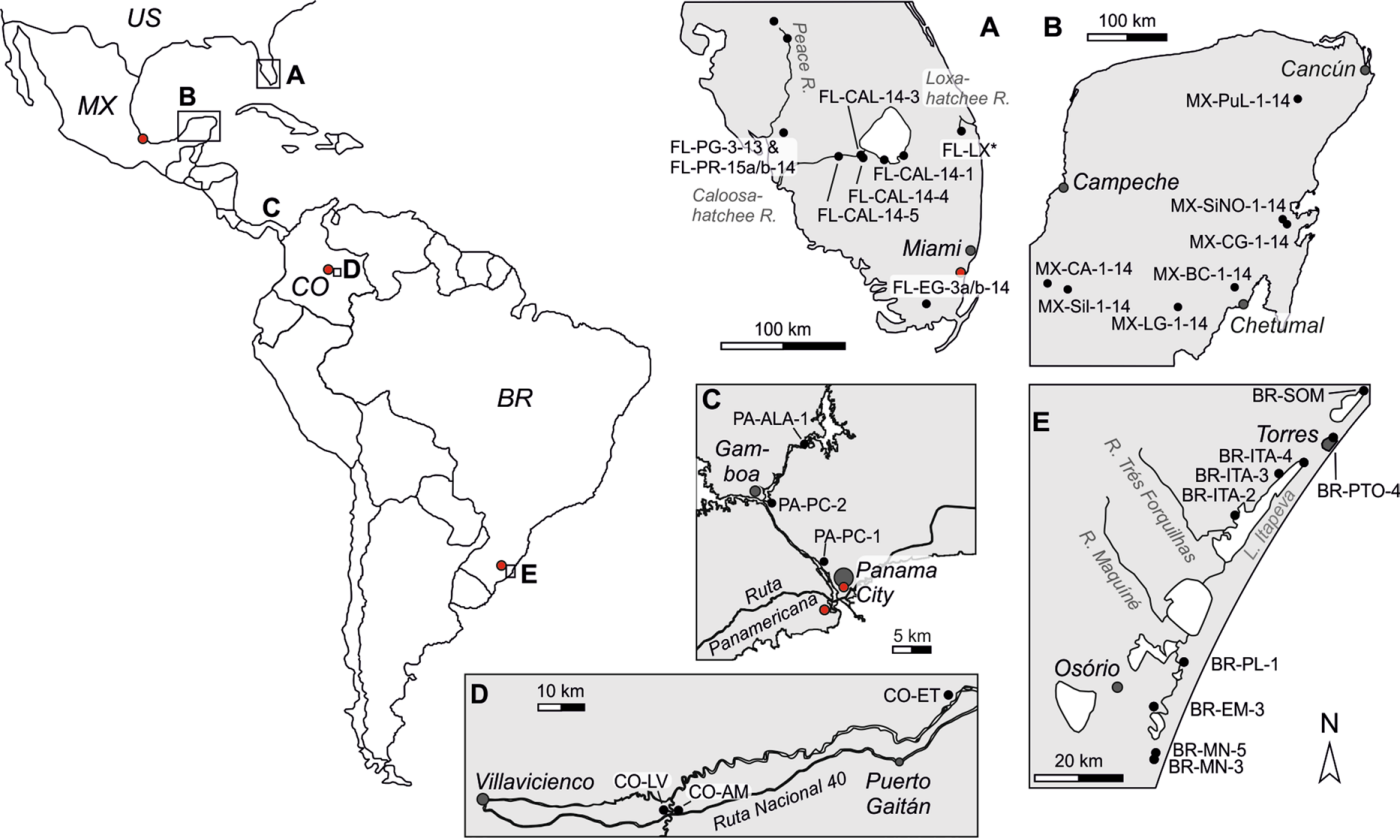
Overview of sampling localities in the study area. Red circles indicate positions of the GNIP stations mentioned in the text. In map A locality FL-LX* refers to samples FL-LX-1 to FL-LX-5

The study areas comprise the known biogeographical range of *C. ilosvayi* from ~ 30° N to ~ 30° S in the Americas. Living ostracodes were sampled in Florida, Mexico (Yucatán), Panama, Colombia, and Southern Brazil (see Fig. 1). The Florida peninsula is a porous plateau of karstic limestone known as the Florida Platform. Much of the peninsula is at or near sea level with a very low relief. Our samples derive from South Florida, which lies within the Atlantic Coastal Plain physiographic province comprising a mixture of flatlands, marshes and swamps as well as the Atlantic coastal ridge running along the Atlantic coast (Long 1974). Like the Florida peninsula the Yucatán is an emergent part of a carbonate platform of Cenozoic limestones. Physiographically, it is therefore very similar to Florida with a low relief and relatively few different geomorphological units (i.e., coastal and karstic plains, and extended karstic and tectono-karst regions; Bautista and Zinck 2010). The major difference to Florida is the absence of surface run off resulting from rapid infiltration (Back and Hanshaw 1970). The dominant features of Panama´s landform are steep and heavily forested mountain ranges and relict volcanoes that form together with volcanoes, drainage basins, several rivers and complex coastlines representing a complex mixture of physiographic regions (Palka 2005). The study area is located within the Canal Zone Lowlands Province which is a region of relatively low topography between the Central Volcanic Cordillera of western Panama and the mountainous Darien isthmus to the east. It encompasses a network of low-gradient river valleys that drain surrounding hills (Marshall 2007). Colombia possesses a high physiographic diversity with coastal, mountainous, and continental areas. The sampling location lies within Orinoquía natural region as part of the Colombian Eastern Plains and belongs to the Orinoco watershed. It is characterized as large low-lying alluvial plains (cf. Vrieling et al. 2002). The Brazilian study area is located at the Atlantic coast in southern Brazilian coastal plain confined by highlands of Paleozoic and Mesozoic sedimentary and volcanic rocks of the Paraná Basin. Samples were derived from barrier-lagoon systems (Tomazelli et al. 2000).

**Fig. 2:**
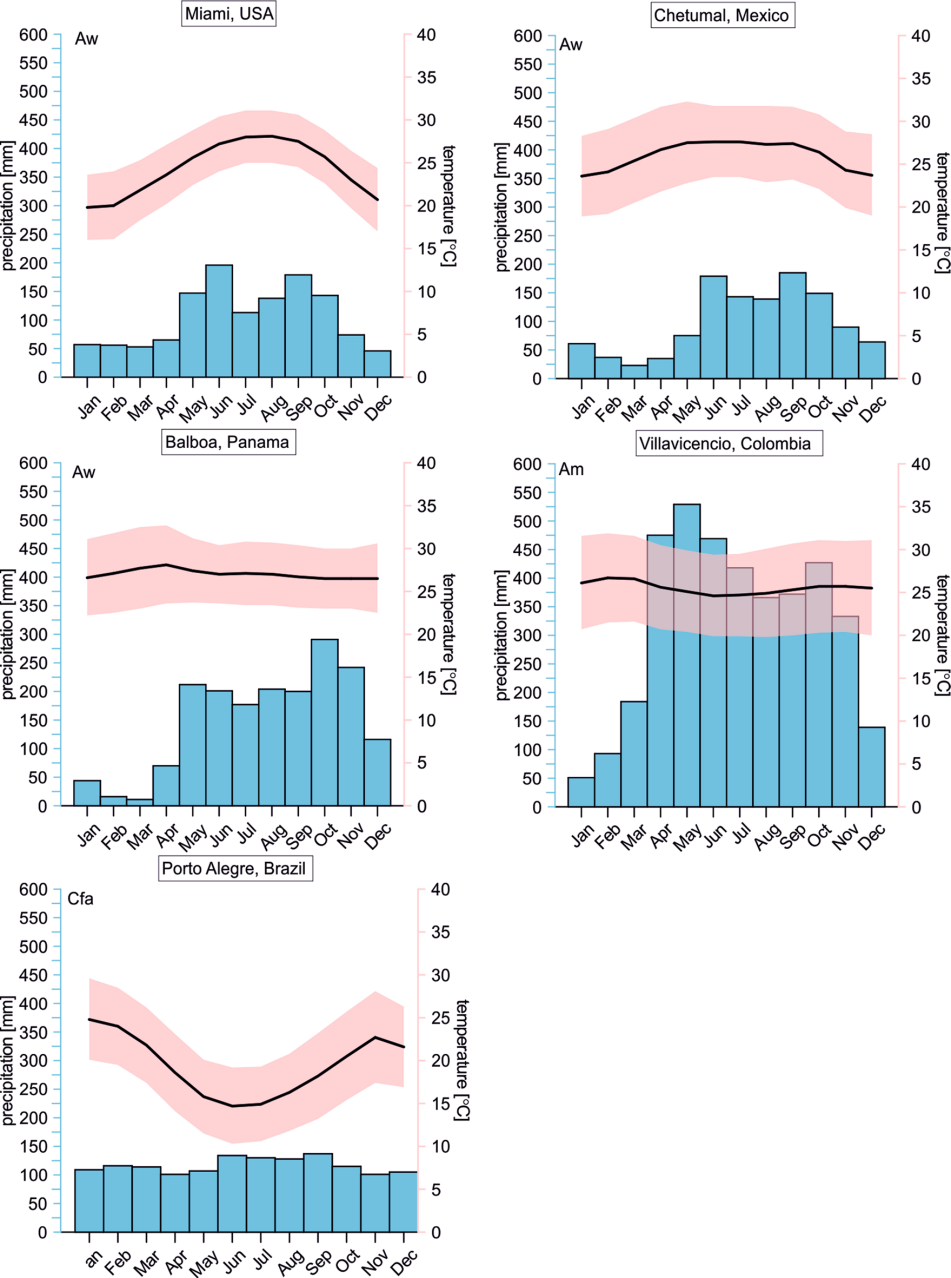
Climatic conditions in the study areas in terms of annual variation of temperature and precipitation (Climate data.org 2019)

According to the Köppen-Geiger climate classification most of the regions are characterized by equatorial climates with a dry winter (Florida, Yucatan, Panama) or with monsoonal precipitation (Colombia). Only southern Brazil possesses a warm temperate climate with perennial precipitation and a hot summer (Kottek et al. 2006). Figure 2 summarizes details on seasonal variation in temperature and precipitation of the regions. Seasonal temperature gradients are more pronounced in the marginal areas of the Neotropics—Florida and Southern Brazil. The temperature difference between the mean highest and lowest monthly temperature amounts up to about 10 °C in Florida and S-Brazil. Central regions such as Colombia and Panama are characterized by nearly constant temperatures throughout the whole year.

Precipitation amount and seasonality (i.e., onset and duration of wet seasons) vary significantly between the regions. Annual precipitation amount is highest in Villavicencio (CL) with 3856 mm a^−1^ followed by Panama City (PA) with 1784 mm a^−1^. Lower and similar amounts occur in Porto Alegre (BR), Miami (USA), and Chetumal (MX) with 1397-, 1267-, and 1180-mm a^−1^, respectively. Due to the large geographical range covered by the study areas, precipitation originates from different moisture sources. Additionally, thunderstorms and tropical cyclones are common during the wet season in the Caribbean region (Florida, Yucatán, Panama) providing large precipitation amounts within hours or a few days (Price et al. 2008). The overall major moisture source for precipitation in South Florida is evaporated water from the ocean southeast of Florida. During the wet season, moisture arises from the trade-wind belt of the tropical North Atlantic. The drier winter season is characterized by alternation of maritime tropical and modified continental polar air from high latitude source. Rarely during the cold season low latitude westerlies bring moisture from the Gulf of Mexico, Caribbean, or even the tropical Pacific (Price et al. 2008). The Caribbean Sea is the dominant moisture source for surface waters in Panama (and Central America) despite climatological evidence that suggests rainfall on the Pacific Coast as being derived from the eastern Pacific Ocean (Lachniet and Patterson 2006). Moisture from the Atlantic Ocean and terrestrial recycling are the most important sources of moisture for Colombia, highlighting the importance of the Orinoco and Amazon basins as regional providers of atmospheric moisture (Hoyos et al. 2018). Recycling of continental precipitation represents the main moisture source of Southern Brazil. Oceanic moisture from the Atlantic Ocean contributes a much smaller part to the annual precipitation amount in Southern Brazil (Martín-Gómez et al. 2016).

## Material and methods

### Sampling

Living *Cytheridella* populations were sampled between 2013 and 2016 in 38 water bodies in South Florida, on the Yucatán Peninsula, Panama, Central Colombia, and South Brazil (Fig. 1). Except one site in Florida (FL-PG-3/FL-PR-15a) sampling took place just once. Study sites encompass different habitats including lakes, wetlands, and rivers. Detailed information is summarized in Table 1. Ostracode material was obtained by sampling of the upper 1–2 cm of the sediment surface with hand nets in littoral zones and shallow still water areas (< 2 m water depth). Field variables (electrical conductivity, water temperature and pH) were measured in situ at all sample sites. Water samples were filtrated using a syringe filter (pore size: 0.45 µm) and stored in 100 ml PE vessels until analysis. Ostracods were picked from sediment samples under a binocular (Zeiss Discovery V8). *C. ilosvayi* was identified by morphological features of the shell in accordance with the description of the appendages by Purper (1974).

**Table 1:** Overview of samples names, locations, sampling date, coordinates and physico-chemical characteristics measured in the field

Sample	Date	Country	Location	N (S)	E (W)	Habitat	Conductivity [µS cm^−1^]	Temperature [° C]	pH	O_2_ [mg/l]
MX-BC-1a	11-08-2014	Mexico	Laguna Bacalar	18° 39ʹ 4.96ʺ	88° 24ʹ 32.48ʺ	Lagoon	2360	31.2	7.5	6.64
MX-SiNo-1a	11-08-2014	Mexico	Siiji No-Ha Cenote	19° 28ʹ 33.5ʺ	88° 03ʹ 15.6ʺ	Cenote	1351	32.2	8	7.82
MX-CA-1	12-08-2014	Mexico	Cenote Azul	18° 48ʹ 43.3ʺ	090° 38ʹ 48.1ʺ	Cenote	575	31	8	8.9
MX-LG-1	12-08-2014	Mexico	Lake "Las Garantias" near Caobas	18° 22ʹ 11.7ʺ	89° 00ʹ 42.0ʺ	Lake	527	31.4	7.8	5.6
MX-Sil-1	12-08-2014	Mexico	Nah Laguna	18° 38ʹ 29.8ʺ	090° 16ʹ 28.1ʺ	Lake	205	32.5	7.8	7.55
MX-PuL-1	13-08-2014	Mexico	Punta Laguna	20° 38ʹ 49.4ʺ	087° 38ʹ 04.1ʺ	Lake	1250	32.1	8.1	7.76
FL-LSS 1a	26-11-2013	USA	Little Salt Spring	27° 4ʹ 29.33ʺ	082° 14ʹ 0.37ʺ	Spring	5110	26.8	7.5	4.95
FL-PG-3	28-11-2013	USA	Hathaway Park	26° 58ʹ 27.04ʺ	081° 53ʹ 21.55ʺ	Upstream estuary	938	20.3	7.9	6.35
FL-BiC	29-11-2013	USA	Big Cypris National Reserve	25° 53ʹ 29.53ʺ	081° 16ʹ 14.52ʺ	Swamp	586	20.8	7.6	n.a
FL-LX-1	31-07-2014	USA	Loxahatchee River	26° 56ʹ 03.0ʺ	080° 10ʹ 36.4ʺ	River	363	30.6	7.3	1.59
FL-LX-2	31-07-2014	USA	Loxahatchee River	26° 56ʹ 32.5ʺ	080° 10ʹ 19.2ʺ	River	375	30.5	7.2	1.82
FL-LX-3	31-07-2014	USA	Loxahatchee River	26° 56ʹ 40.28"	80° 10ʹ 15.94"	River	375	31.7	n.a	1.8
FL-LX-5	31-07-2014	USA	Loxahatchee River	26° 56ʹ 49.8ʺ	080° 10ʹ 12.4ʺ	River	375	30.4	7.1	2.2
FL-EG-3	02-08-2014	USA	Rock Reef Pass Trail, Everglades National Park	25° 26ʹ 2.0ʺ	080° 45ʹ 12.3ʺ	Dwarf cypress marsh	189	33.1	8.1	9.9
FL-CAL-14-1	06-08-2014	USA	Lake Okeechobee at Belle Glade	26° 43ʹ 37.9ʺ	080° 42ʹ 10.5ʺ	Lake	444	31	8.6	–
FL-CAL-14-3	06-08-2014	USA	Okeechobee Canal	26° 50ʹ 22.4ʺ	081° 04ʹ 51.8ʺ	Channel	238	34	6.7	2.46
FL-CAL-14-2	06-08-2014	USA	Lake Okeechobee canal at Clewiston	26° 45ʹ 41.5ʺ	080° 55ʹ 11.7ʺ	Lake	676	30.5	7.4	3.9
FL-CAL-14-4	06-08-2014	USA	Alwin Ward Public Boat Ramp-Caloosahatchee Launch lane	26° 50ʹ 09.8ʺ	081° 05ʹ 14.4ʺ	River	724	35.5	7.5	5.3
FL-CAL-14-5	06-08-2014	USA	Ortona Beach Ramp	26° 47ʹ 21.7ʺ	081° 18ʹ 33.6ʺ	River	550	34.7	7.4	8.4
FL-PR-1	07-08-2014	USA	Lake Hancock Boat Ramp	28° 0ʹ 7.31"	81° 51ʹ 4.22"	River	247	28.3	7	3.02
FL-PR-6	07-08-2014	USA	Peace River Heritage Homeland Boat Ramp	27° 48ʹ 46.2ʺ	081° 47ʹ 36.9ʺ	River	189	28.3	6.5	2.35
FL-PR-15a	08-08-2014	USA	Hathaway Park	26° 58ʹ 26.99"	81° 53ʹ 21.81"	Artificial river branch	297	31.2	7.1	4.28
BR-PL-1-15	03-09-2015	Brazil	Passos da Lagoa	29° 51ʹ 16.1ʺ	050° 06ʹ 57.9ʺ	Lagoon	70	22.2	7.3	7.3
BR-EM-3-15	03-09-2015	Brazil	Emboaba Lagoon	29° 57ʹ 52.8ʺ	050° 13ʹ 27.4ʺ	Lagoon	49.3	20.2	6.8	8.7
BR-MN-3-15	04-09-2015	Brazil	Rio de Relógio	30° 04ʹ 10.3ʺ	050° 12ʹ 20.8ʺ	Channel	149.2	18.7	7.4	8.38
BR-MN-5-15	04-09-2015	Brazil	Rio de Relógio	30° 02ʹ 45.7ʺ	050° 11ʹ 09.9ʺ	Channel	51.6	19.2	6.5	8.63
BR-SOM-1-15	05-09-2015	Brazil	Sombrio	29° 07ʹ 44.4ʺ	049° 38ʹ 18.4ʺ	Lagoon	79.9	19	6.4	5.84
BR-RMA-3-15	05-09-2015	Brazil	Rio Mampituba	29° 17ʹ 28.9ʺ	049° 46ʹ 43.3ʺ	Channel	62.3	21.5	6.1	6.3
BR-ITA-2-15	06-09-2015	Brazil	Itapeva Lagoon	29° 29ʹ 48.3ʺ	049° 57ʹ 43.9ʺ	Lagoon/marsh	106	20.7	6.3	9.63
BR-ITA-3-15	06-09-2015	Brazil	Itapeva Lagoon	29° 23ʹ 35.0ʺ	049° 50ʹ 28.9ʺ	Lagoon	93.1	19.2	6.4	6.72
BR-ITA-4-15	06-09-2015	Brazil	Itapeva Lagoon	29° 22ʹ 32.6ʺ	049° 47ʹ 39.2ʺ	Lagoon	61.5	20.4	6.3	8.79
BR-PTO-4-15	07-09-2015	Brazil	Passos de Torres Lagoa	29° 18ʹ 39.6ʺ	049° 42ʹ 32.8ʺ	Lagoon	279	19.6	6.8	9.03
CO-ET-1-15a	04-02-2015	Colombia	Estero Texas	04° 24ʹ 31.2ʺ	071° 58ʹ 44.5ʺ	Spillway channel, permanent flooded	21.4	32	6.7	7.35
CO-AM-1-15	05-02-2015	Colombia	Alto Menegua	04° 06ʹ 08.8ʺ	072° 54ʹ 48.1ʺ	Stream	20.5	27.2	6	7.69
CO-LV-1-15	06-02-2015	Colombia	Laguna La Venturosa	04° 05ʹ 57.7ʺ	072° 57ʹ 55.4ʺ	Lake	12.2	30.8	7.6	7.35
PA-ALA-1-16	25-07-2016	Panama	Lago Alajuela	09° 12ʹ 50,2ʺ	079° 37ʹ 04.2ʺ	Lake (dammed lake)	140.7	28.2	7.8	-
PA-PC-1-16	25-07-2016	Panama	Panama Canal	09° 00ʹ 41.1ʺ	079° 35ʹ 43,0ʺ	Lake	1097	28.4	7.6	-
PA-PC-2-16	25-07-2016	Panama	Gamboá	09° 06ʹ 48.4ʺ	079° 41ʹ 27.3ʺ	River (mouth area)	165	27	7.9	-

### Chemical and isotopic analyses of the water

The chemical composition as well as the isotopic composition of the sampled water (δ^18^O_water_, δD_water_) and dissolved inorganic carbon (δ^13^C_DIC_) were measured at the laboratory center of JR-AquaConSoL in Graz. The analytical procedure for measurements of δ^18^O_water_, δD_water_ that was used in this study is similar to the method described by Brand et al. (2009). The classic CO_2_–H_2_O equilibrium technique (Epstein and Mayeda 1953) with a fully automated device adapted from Horita et al. (1989) coupled to a Finnigan DELTAplus Dual Inlet Mass Spectrometer was used for the measurement of oxygen isotope distribution. The stable isotopes of hydrogen of the water molecule were measured using a Finnigan DELTAplus XP mass spectrometer working in continuous flow mode by the chromium reduction method (Morrison et al. 2001). Isotopic composition of DIC was analyzed using a Gasbench II device (Thermo) connected to a Finnigan DELTAplus XP isotope ratio mass spectrometer comparable to setups in other studies (Spötl 2005). Results of isotopic measurements are given in per mil (‰) with respect to Vienna Standard Mean Ocean Water (V-SMOW) and Vienna Peedee Belemnite (V-PDB), respectively, using the standard delta notation. The analytical precision for stable isotope measurements is ± 0.8‰ for δD_water_, ± 0.08‰ for δ^18^O_water_ and ± 0.1‰ for δ^13^C_DIC_ values.

Concentrations of dissolved components in the water samples were measured using ion chromatography (Dionex IC S 3000) with an analytical precision of ± 3%. The total alkalinity of the solutions was determined by titration using a 0.02 M HCl solution with an analytical precision of ± 2%. The aqueous speciation of the water, ion activities and saturation index in respect to calcite were calculated using the PHREEQC computer code (Parkhurst and Appello 1999) with its minteq.v4 data-base.

### Isotopic analyses of *C. ilosvayi*

Stable isotopic measurements of ostracodes were carried out at the Institute of Earth Sciences, University of Graz, GeoZentrum, University of Erlangen, and Institute of Geophysics and Geology, University Leipzig. Per sample 1–16 measurements were performed for carbon and oxygen stable isotopes containing two to eight valves of *C. ilosvayi* (female, male and A-1) depending on the valve size and if fragments were missing. Adult and juvenile valves were analysed separately. Prior to isotopic analyses soft part tissues and contaminations were removed from all ostracod valves with deionized water, brushes and entomological needles. If necessary, single valves were cleaned with H_2_O_2_ (10%) for five to ten minutes at room temperature.

The shells were reacted with 100% phosphoric acid at 70 °C in a Kiel II automated reaction system and measured with a Finnigan DELTA^plus^ isotope-ratio mass spectrometer. Reproducibility of replicate analyses for standards (in-house and NBS 19) was better than ± 0.08‰ for δ^13^C_ostracodes_ and ± 0.1‰ for δ^18^O_ostracodes_. All carbonate isotopic values are quoted relative to V-PDB.

### Seasonal oxygen isotopic composition of the water bodies

In order to provide a reference for ostracode oxygen isotopic compositions, the isotopic composition of water and the temperature dependent oxygen isotope fractionation between calcite and precipitating water according to the relationship of Coplen (2007) were used to calculate the δ^18^O_calcite eq_ value at isotope equilibrium. Since ostracodes grow by molting, which lasts only hours to a few days (Turpen and Angell 1971), it can be assumed that the transition from one developmental stage to another (e.g., A-1 to adult specimens) occurs within a short period (days up to a few weeks; e.g., Havel and Talbott 1995; Morin and Gerrish 2008).

Meyer et al. (2017b) whose study is based on ostracode and water samples from southern Florida have shown calcification of *Cytheridella* to be restricted to early spring, where assumptions prior to the comparison between δ^18^O_ostracode_ and δ^18^O_calcite_eq_ included: (1) changes in meteoric origin are the major control on δ^18^O_water_ of the precipitating water (cf. Henderson and Shuman 2009; Lachniet and Patterson, 2009); (2) evaporation is seasonally constant and of minor importance for lotic water bodies (Gremillion and Wanielista 2000); (3) the variation in δ^18^O_ostracode_ values is environmentally (i.e., temperature, δ^18^O_water_) induced; (4) the last molting period for the entire population lasts 1 month at maximum; (5) *Cytheridella* provides a constant positive vital effect of ~  + 1‰ (Escobar et al. 2012) for its oxygen isotope composition in comparison to oxygen isotope equilibrium conditions given by Coplen (2007); (6) the correction of the δ^18^O_meteoric_ with lake water δ^18^O values is sufficient to emulate a specific calcite precipitated in both lotic and lentic water bodies.

**Fig. 3:**
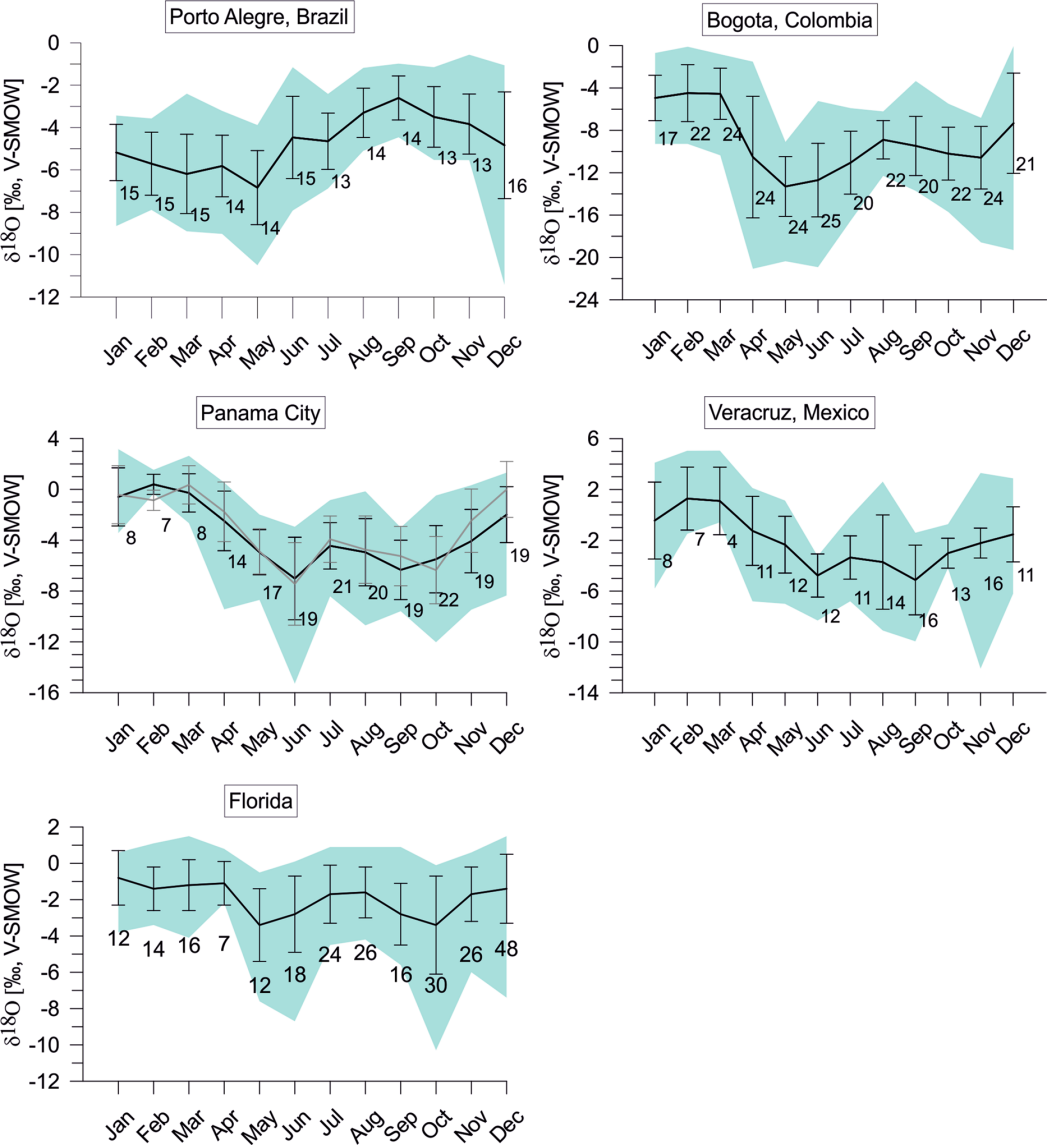
Monthly variation of oxygen isotope distribution (δ^18^O_water_) in precipitating meteoric water in the study areas. Data were obtained from the global network of isotopes in precipitation (GNIP) database. Displayed are mean values (black line) with its standard deviation and minimum and maximum values (green area). Numbers indicate the measurements available for the respective months. For Panama two data sets are displayed for station Panama University (black line) and Howard (grey line)

In order to follow the approach by Meyer et al. (2017b) applied to Floridian *Cytheridella,* we used monthly means, minima, and maxima of temperature (T_mean_, T_min_, T_max_) and δ^18^O_water_ (δ^18^O_max_, δ^18^O_min_, δ^18^O_mean_) from local meteoric datasets to estimate individual expected δ^18^O (δ^18^O_calcite_ex_) values under conditions of isotopic equilibrium in order to generate a reference data set to which measured δ^18^O_ostracodes_ could be compared. Temperature and meteoric data were obtained from Climate-Data.org (2019) for the cities of Miami (Florida), Chetumal (Mexico), Panama City (Panama), Villavicencio (Columbia), and Porto Alegre (Brazil) (see Fig. 2). The δ^18^O_water_ values were received from the Global Network for Isotopes in Precipitation (GNIP). The locations of the GNIP stations are displayed in Fig. 1. Seasonal distribution of δ^18^O_water_ is displayed in Fig. 3.

As proposed by Meyer et al. (2017a) a correction of δ^18^O_meteoric_ values from the GNIP database for every water body is done by the difference between δ^18^O_water_ of the water sample and the δ^18^O_meteoric_ of the respective sample month according to the equation

1 $$\Delta^{{{18}}} {\text{O}}_{{{\text{m}} - {\text{w}}}}^{*} = \delta^{{{18}}} {\text{O}}_{{{\text{meteoric}}}} \left( {{\text{measured}};{\text{ GNIP database}}} \right) \, - \delta^{{{18}}} {\text{O}}_{{{\text{water}}}} \left( {\text{measured herein}} \right)$$

To yield the corrected oxygen isotope composition of the individual water body throughout the year: δ^18^O_water_^*^ = δ^18^O_meteoric_—Δ^18^O_m-w_^*^. If possible, literature data of lake water δ^18^O were used to receive further oxygen isotope compositions for the respective locality. In Colombia the only GNIP station is in Bogota lies at an altitude of 2640 m a. s. l. in contrast to Villavicencio that lies at 467 m a. s. l. Since it is known that there is an empirically consistent and linear relationship between change in elevation and change in the isotopic composition of meteoric precipitation along altitudinal transects with ~ 0.2‰/100 m decrease in the isotopic composition of meteoric precipitation with increasing elevation (Rozanski and Araguás-Araguás 1995) we used also an altitude-corrected δ^18^O_meteoric_ for the calculation of the calcite in order to test the validity of the lake water-based corrections. In cases where water sample values were not available for the locality itself (e.g., FL-CAL-3; BR-ITA-2 and 3) average values were calculated from the next sample sites within the water body. In order to display the effect of the correction, the site-specific δ^18^O_eq_ex_ ranges are displayed together with the more general uncorrected calcites.

The difference in δ^13^C between water and calcite is calculated by

2 $$\Delta^{{{13}}} {\text{C}}_{{{\text{Cc}} - {\text{DIC}}}} = \delta^{{{13}}} {\text{C}}_{{{\text{ostracode}}}} {-}\delta^{{{13}}} {\text{C}}_{{{\text{DIC}}}}$$

## Results

### Hydrochemical classification

**Fig. 4:**
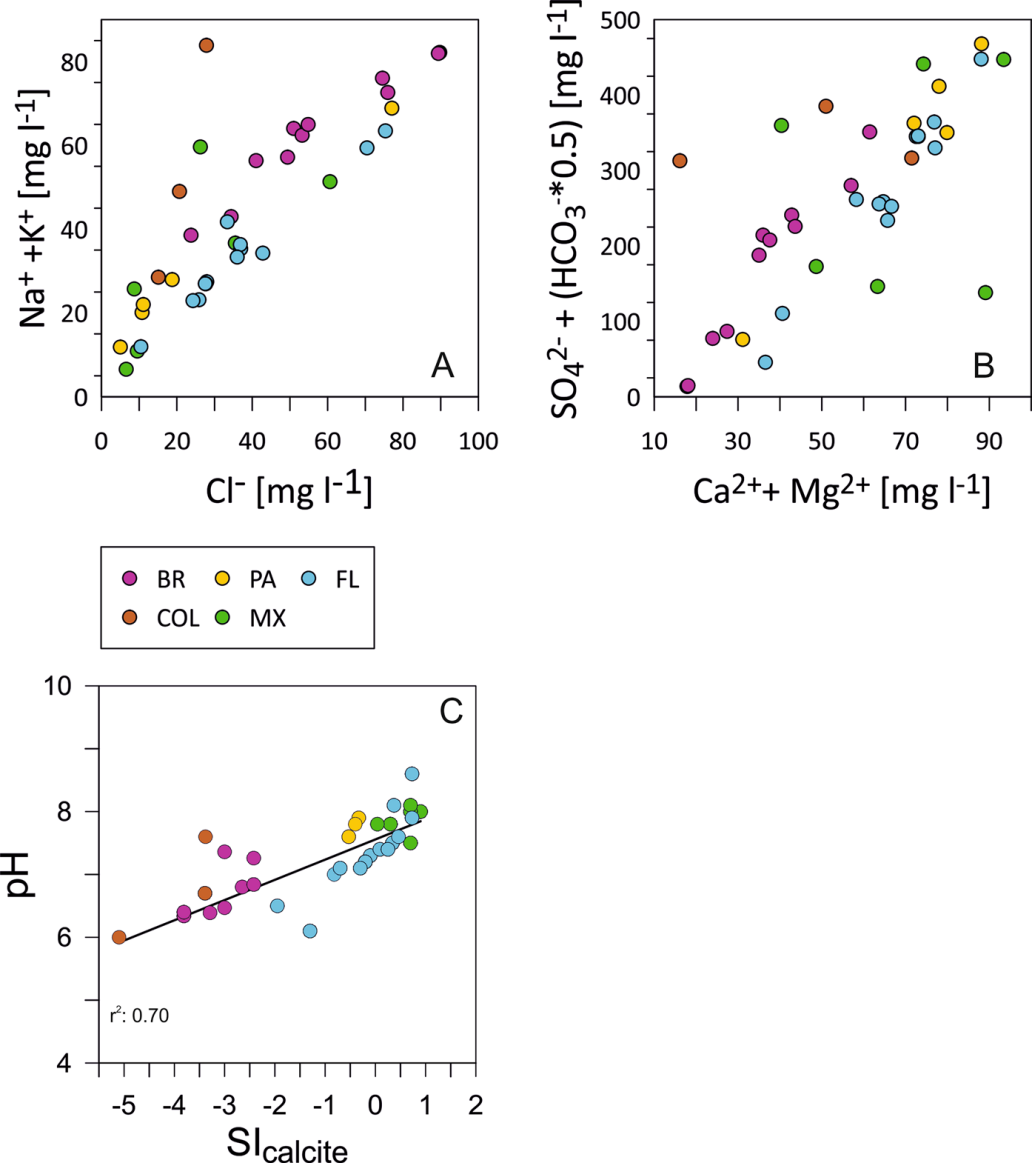
Hydrochemical characteristics in terms of concentration displayed for **A** Na^+^ (+ K^+^) and Cl^−^, **B** SO_4_^2−^ + HCO_3_^−^ and Ca^2+^  + Mg^2+^ and regression plot of pH vs. SI_calcite_. Colour codes refer to regions Florida (FL), Mexico (MX), Brazil (BR), Panama (PA), and Colombia (CO)

The investigated sites provided freshwater to moderately saline waters with electric conductivity values ranging from 12 to 5110 µS cm^−1^, representing the total ion concentrations dissolved in the water. Mean conductivity values of the regions differ strongly; with the highest mean of 1045 µS cm^−1^ in Mexico followed by Florida with 729 µS cm^−1^. Panama and Southern Brazil provide means of 468 and 100 µS cm^−1^, respectively. The lowest mean conductivity is found for Colombia with 49 µS cm^−1^. For details see Table 1. Measured pH values range from 6.0 to 8.6, whereas the majority of samples provide values between 6.7 and 7.8. The regions provide mean pH values from high to low in the following order: Mexico (7.9) ≥ Panama (7.8) ≥ Florida (7.4) ≥ Colombia (6.8) ≥ Brazil (6.6). Measured water temperatures range from 18.7 to 35.5 °C. Lowest water temperature was measured in Brazil. Highest mean temperature is documented in Mexico with 31.7 °C, followed by Colombia and Florida with about 30 °C, respectively. Panama and S-Brazil show temperature means of 27.9 and 20.1 °C, respectively. Concentrations of Na^+^ (+ K^+^) and Cl^−^ are lowest in Mexico, Panama, and Colombia. They vary strongly in Florida, but provide generally mean concentrations compared to the other regions. Highest concentration of sodium and chloride are shown by Brazil. The picture is almost reverse for SO_4_^2−^ with HCO_3_^−^ compared to Mg^2+^ and Ca^2+^ (Fig. 4). Brazil is characterized by variable but generally lower to lowest concentrations of Mg^2+^ and Ca^2+^ followed by Florida, Mexico, and Colombia. Highest concentrations of Mg^2+^ and Ca^2+^ and SO_4_^2−^ and HCO_3_^−^ occur in solutions of Panama. Detailed major anion and cation compositions are displayed in the piper diagram provided as supplementary material (Supplementary Fig. 1).

The waters of the different regions diverge also for the saturation index with respect to calcite expressed by SI_calcite_. The SI_calcite_ range covers values from − 5.1 (strongly undersaturation; in Columbia) up to 0.9 (highest supersaturation, in Mexico). The solutions of Panama, Columbia, and Brazil are all undersaturated with calcite and show a gradient with relatively low undersaturation of Panama (mean SI_calcite_: − 0.4) followed by relatively strong undersaturation of Brazilian and Columbian samples (mean SI_calcites_: − 3.1 and − 3.6, respectively). Floridian solutions provide positive (Caloosahatchee River) as well as negative (Peace River, Loxahatchee River) SI_calcite_-values with a very low mean of -0.08. Only Mexican waters are all saturated with respect to calcite and provide a relatively high mean SI_calcite_ of 0.7.

### Isotopic composition of analyzed local water

**Fig. 5:**
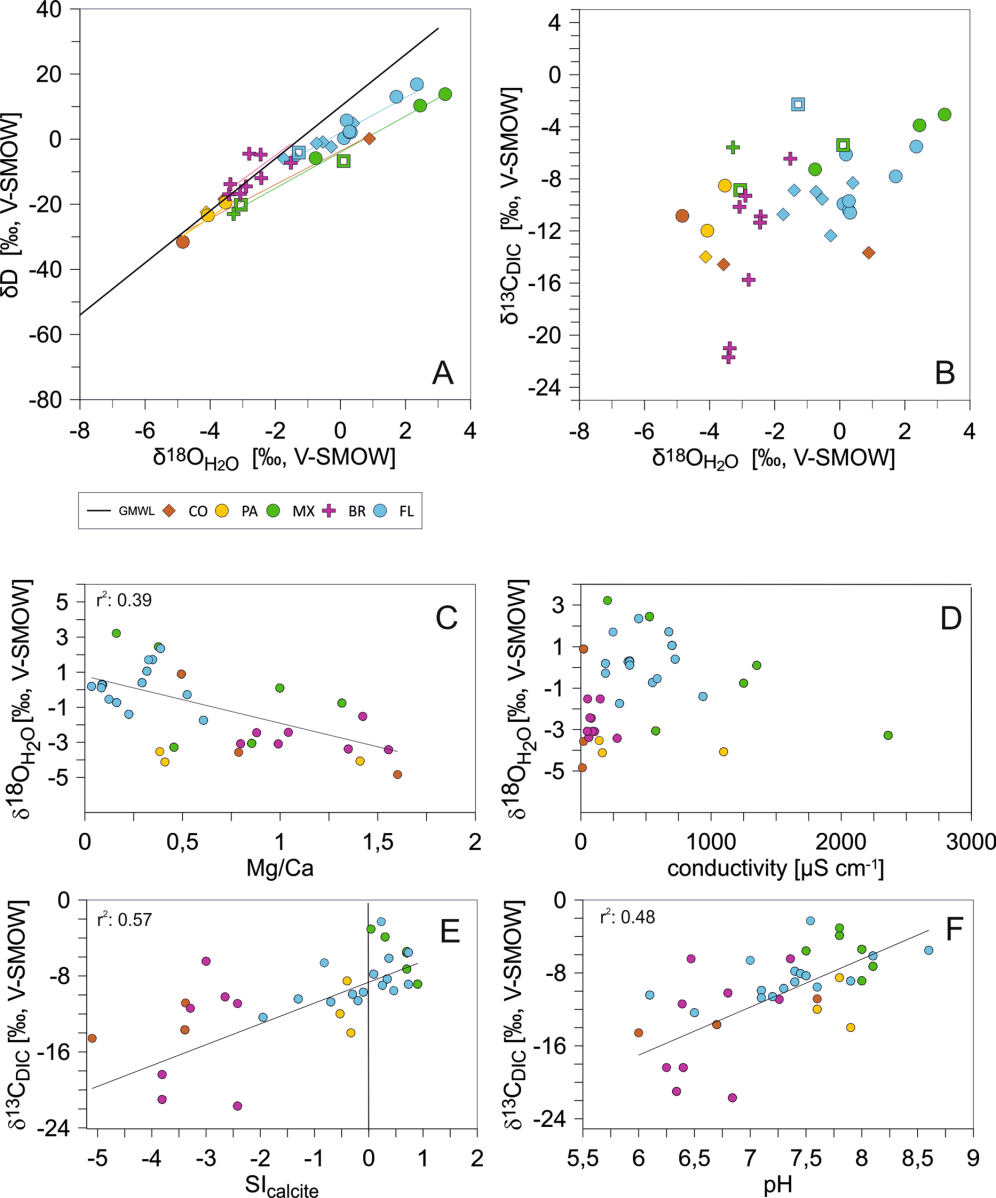
**A** Stable hydrogen and oxygen isotope distribution of water in comparison to the global meteoric water line (GMWL). Coloured lines represent regression lines of the samples representing evaporation trends. **B** Oxygen isotope composition of the water vs. stable carbon isotopes of dissolved inorganic carbon (DIC). **C** Oxygen isotope composition of the water vs. molar aqueous Mg/Ca ratio. **D** Oxygen isotope composition of the water vs. electric conductivity. **E** Stable carbon isotope distribution in DIC vs. saturation index in respect to calcite. **F** Stable carbon isotope distribution in DIC vs. pH value. Colour codes refer to regions Florida (FL), Mexico (MX), Brazil (BR), Panama (PA), and Colombia (CO)

The isotopic values of the water samples range from − 31.6 to 16.9‰ for δD (VSMOW), from − 4.8 to 3.2‰ for δ^18^O (VSMOW), and from − 21.7 to − 2.3‰ for δ^13^C_DIC_ (VPDB). Regional means and ranges are summarized in Table 2. Samples from Florida and most of the samples from Mexico provide a negative deviation to the global meteoric water line (GMWL) and describe local evaporation lines with Florida: δD = 5.44 * δ^18^O + 1.94, and Mexico: δD = 5.53 * δ^18^O − 4.03. With one exception of a river sample from Colombia, the samples from Panama, Colombia, and S-Brazil fall on the GMWL trend, independently to the habitat type (see Fig. 5A). The cross-plot of oxygen and carbon isotope values (Fig. 5B) shows that rivers and lakes differ widely in their δ^18^O values while δ^13^C variations are relatively small. Coastal lagoons display strong variations in δ^13^C and only minor differences in δ^18^O. The oxygen isotope composition of the solutions shows a weak correlation with Mg/Ca (r^2^: 0.39) and no correlation with conductivity (r^2^ ≤ 0.05) (Fig. 5C, D). Carbon isotopes of the solutions (δ^13^C_DIC_) are correlated (r^2^: 0.57) with the calcite saturation index (Fig. 5E). Thus, solutions which are saturated with respect to calcite provide the most positive δ^13^C values. A slightly lower correlation (r^2^: 0.48) is revealed for carbon isotopes of DIC and pH.

**Table 2:** Stable oxygen and carbon isotope (δ^18^O_ostracode_, δ^13^C_ostracode_) data of ostracode calcite of and isotopic data (δD, δ^18^O_H2O_, δ^13^C_DIC_) of corresponding water samples

Sample	Water			δ^18^O_ostracode_					δ^13^C_ostracode_				
	δ^18^O	δ^13^C	Temp	n	Mean	Min	Max	Stdv	Mean	Min	Max	Stdv	
PG-3	− 1.4	− 8.88	20.3	8	− 1.80	− 3.05	− 0.53	0.90	− 8.17	− 9.59	− 6.64	0.92	a
BiC-1	− 0.54	− 9.55	20.8	2	− 1.27	− 1.29	− 1.26	–	− 9.04	− 10.31	− 7.76	–	a
LSS-1	− 1.28	− 2.28	26.8	1	− 2.09	–	–	–	-2.61	–	–	–	a
FL-LX-1	0.28	− 9.7	30.6	8	− 0.22	− 1.78	0.58	0.72	− 7.95	− 8.71	− 7.04	0.61	a
FL-LX-2	0.32	− 10.6	30.5	9	− 1.26	− 2.24	− 0.27	0.63	− 8.51	− 9.24	− 7.77	0.43	a
FL-LX-3	0.28	− 10.42	31.7	8	− 0.76	− 1.54	0.55	0.63	− 8.44	− 9.47	− 7.96	0.50	a
FL-LX-5	0.11	− 9.92	30.4	7	− 1.11	− 2.87	0.13	1.07	− 8.21	− 8.75	− 7.29	0.56	a
FL-EG-3	0.19	− 6.13	33.1	7	− 1.30	− 2.42	0.66	1.23	− 6.01	− 7.87	− 2.71	1.96	a
FL-CAL-14-1	2.35	− 5.52	31.0	4	1.25	0.78	1.82	0.43	− 6.27	− 6.53	− 6.06	0.19	a
FL-CAL-14-2	1.72	− 7.81	30.5	6	0.05	− 0.68	0.53	0.41	− 6.08	− 6.34	− 5.69	0.22	a
FL-CAL-14-3	–	–	34.0	29	1.45	− 2.30	2.48	1.18	− 6.72	− 10.51	− 5.54	1.16	c
FL-CAL-14-4	0.4	− 8.31	35.5	16	1.12	− 0.16	2.28	0.74	− 7.03	− 7.86	− 6.24	0.49	a
FL-CAL-14-5	− 0.73	− 8.98	34.7	8	− 1.03	− 2.28	0.30	1.09	− 8.06	− 8.52	− 7.38	0.43	a
FL-PR-6	− 0.28	− 12.36	28.3	4	− 1.02	− 1.25	− 0.89	0.13	− 9.10	− 9.42	− 8.66	0.28	a
FL-PR-15	− 1.74	− 10.73	31.2	7	− 2.09	− 2.99	− 0.95	0.67	− 8.77	− 9.59	− 8.18	0.48	a
MX-SiNo	0.1	− 5.41	32.2	12	− 1.24	− 4.61	1.05	1.69	− 6.39	− 8.57	− 5.24	1.17	
MX-BC	− 3.28	− 5.58	31.2	11	− 4.59	− 5.23	− 3.52	0.51	− 4.43	− 5.17	− 3.67	0.48	
MX-LG	2.45	− 3.89	31.4	6	1.71	0.61	2.34	0.77	− 3.26	− 4.59	− 2.83	0.71	
MX-Sil	3.22	− 3.06	32.5	3	1.38	1.36	1.39	−	− 2.05	− 2.50	− 1.75	–	
MX-CA	− 3.06	− 8.86	31.0	23	− 4.90	− 5.39	− 4.12	0.29	− 8.29	− 9.76	− 7.15	0.61	
MX-PuL	− 0.76	− 7.27	32.1	9	− 3.10	− 3.24	− 3.00	0.08	− 7.52	− 8.49	− 6.81	0.58	
CO-ET	0.89	− 13.67	32.0	7	− 2.42	− 4.38	− 0.96	1.17	− 14.86	− 15.41	− 14.13	0.38	c
CO-AM	− 3.57	− 14.57	27.2	1	− 6.24	–	–	–	− 13.91	–	–	–	c
CO-LV	− 4.84	− 10.84	30.8	1	− 6.93	–	–	–	− 7.89	–	–	–	c
BR-EM-3	− 3.08***	− 10.2 ***	20.2	5	− 2.50	− 3.29	− 0.70	0.98	− 11.98	− 12.60	− 10.55	0.73	b,c
BR-PL	− 2.43	− 10.9	22.2	3	− 3.08	− 3.41	− 2.76	–	− 9.66	− 9.75	− 9.52	0.10	c
BR-MN-3	− 1.52*	− 6.45*	18.7	3	− 1.47	− 1.86	− 1.10	–	− 5.44	− 8.05	− 3.66	–	c
BR-MN-5	− 1.52*	− 6.45*	19.2	2	− 1.07	− 2.03	− 0.11	–	− 6.93	− 7.89	− 5.97	–	c
BR-SOM	− 2.45	− 11.4	19.0	3	− 3.28	− 4.92	− 2.37	–	− 10.79	− 11.13	− 10.56	–	b
BR-ITA-2	–	–	20.7	2	− 2.83	− 3.35	− 2.31	–	− 5.08	− 5.53	− 4.64	–	c
BR-ITA-3	–	–	19.2	10	− 2.14	− 2.53	− 1.88	0.19	− 2.32	− 5.73	0.44	2.09	c
BR-ITA-4	− 3.38	− 21	20.4	9	− 3.59	− 3.90	− 3.31	0.21	− 10.79	− 11.65	− 9.20	0.78	b
BR-PTO-4	− 3.1**	− 11.23**	19.6	10	− 2.82	− 3.25	− 2.09	0.32	− 10.6	− 11.46	− 9.77	0.52	c
PA-PC-1	− 4.07	− 11.98	28.4	3	− 5.44	− 5.67	− 5.22	–	− 11.11	− 11.56	− 10.7	–	c
PA-PC-2	− 4.12	− 13.99	27.0	2	− 5.29	− 5.5	− 5.08	–	− 12.16	− 12.61	− 11.7	–	c
PA-ALA-1	− 3.53	− 8.52	28.2	2	− 4.95	− 4.97	− 4.94	–	− 9.12	− 9.61	− 8.63	–	c

*n* number of measurements

*Isotopic values from MN-1

**Isotopic values from PTO-3

***Isotopic values from EM-1

^a^Published in Meyer et al. (2017a, b)

^b^Measurements Univ. Erlangen

^c^Measurements Univ. Leipzig

### Isotopic characteristics of *C. ilosvayi*

**Fig. 6:**
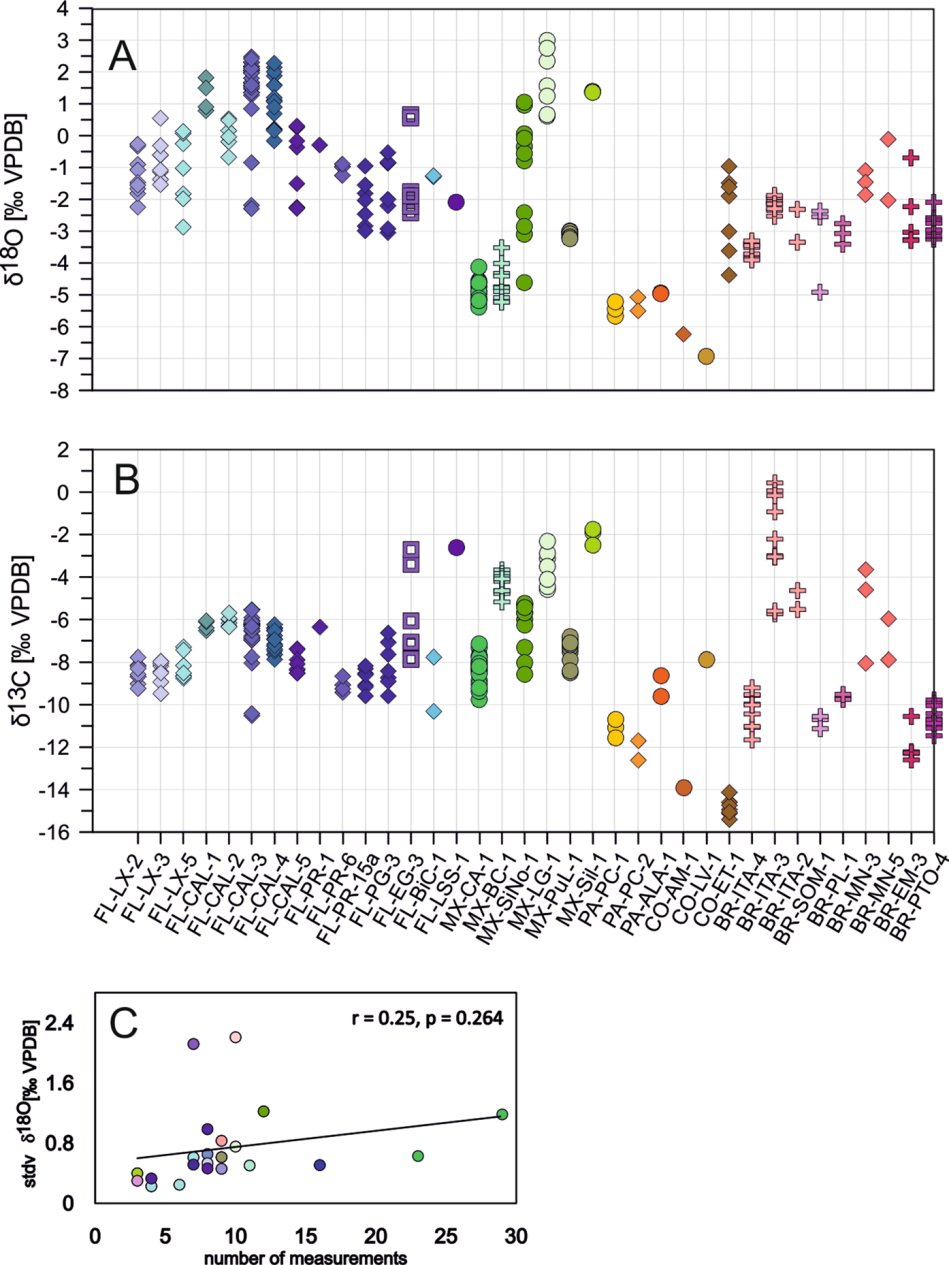
Stable oxygen (**A**) and carbon isotope variation (**B**) of ostracode valves in the samples and relationship between sample size and standard deviation of oxygen isotopes (**C**). Symbols refer to different habitat types including rivers, canals (diamonds), wetlands (square), lakes (circles), and coastal lagoons (crosses)

**Fig. 7:**
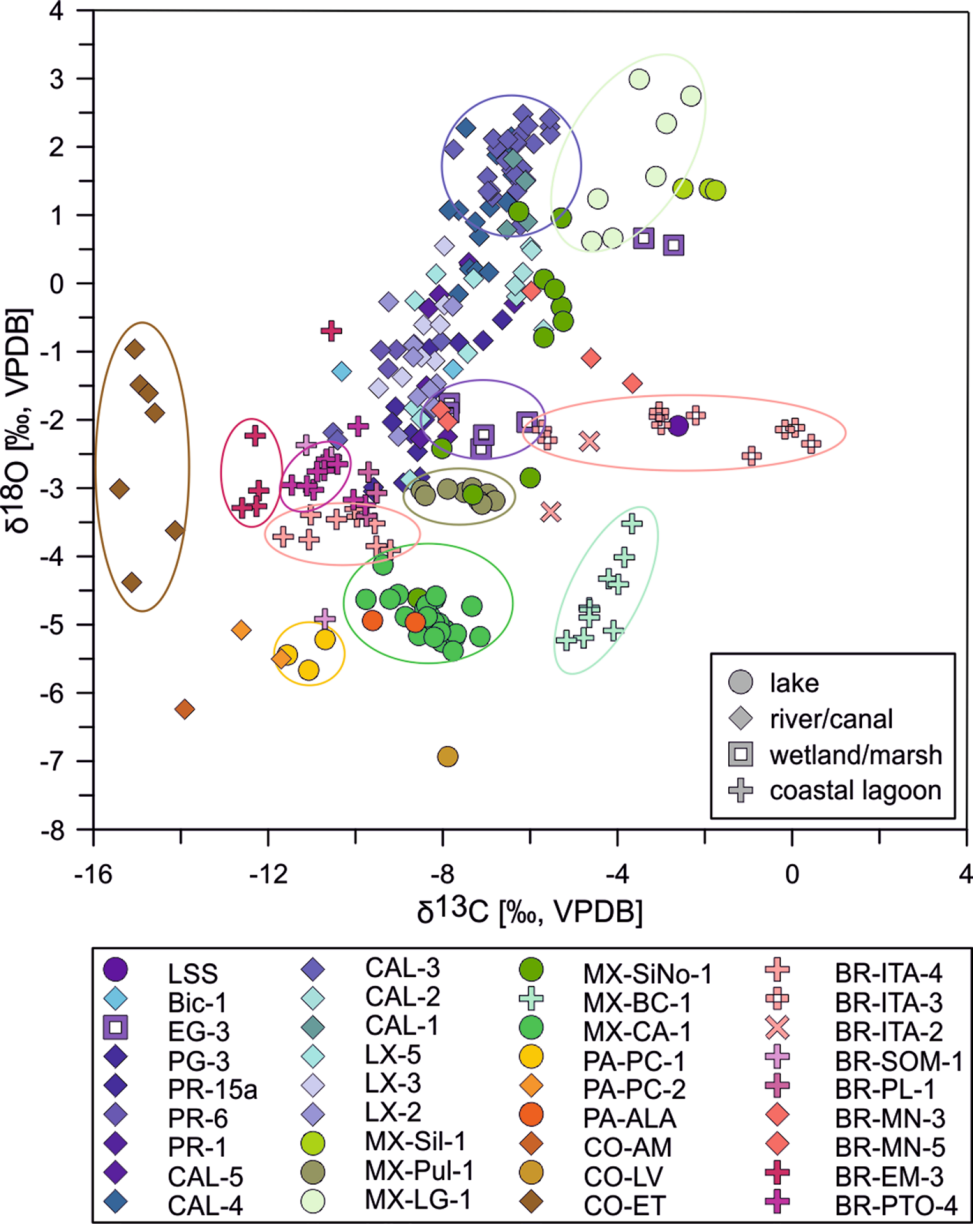
Cross plot of stable oxygen and carbon isotopes of ostracode valves of all samples

Oxygen and carbon isotope values of *Cytheridella* range from − 6.93 to 2.99‰ for δ^18^O (VPDB), and from − 15.41 to 0.44‰ (VPDB) for δ^13^C. Minimum values of oxygen and carbon isotopes are revealed by samples from Colombia (CO-LV, CO-ET). Maximum values are provided for carbon by Brazil (BR-ITA-3) and for oxygen by Mexico (MX-LG). Variation ranges and means differ for both oxygen and carbon on a regional scale. Generally higher oxygen values (~ − 3 to + 3‰) are exhibited by Floridian samples and some samples from Mexico. Brazilian samples and one sample from Colombia provide lower or intermediate values (− 5 to 0‰) and lowest values are provided by samples from Panama and remaining Colombia samples (− 7 to − 5‰). Regional carbon isotopes show a different pattern. While Florida, Mexico and Brazil show relatively similar means; variation ranges between samples differ relatively strongly. Especially Brazilian material shows pronounced differences between the samples. It must be noted that a very strong range in δ^13^C occurs within a water body in BR (BR-ITA; see Fig. 6). Generally low values are shown by Colombian and Panamanian samples (≤ − 8‰). There is a weak positive relationship (r = 0.25, p = 0.264) between the number of measurements and the standard deviation of the sample. Figure 7 compares the oxygen and carbon isotope composition of all analyzed specimens in consideration of the water body type. There can be seen that rivers and most of the lakes show greater variations in δ^18^O whereas δ^13^C values vary on relatively small ranges. Specimens from Brazilian coastal lagoons exhibit the opposite trend with relatively large variation ranges in δ^13^C and small variation ranges in δ^18^O. The Mexican lagoon MX-BC sample shows a similar δ^18^O and δ^13^C trend as rivers and streams; strong variation in δ^18^O and small ranges in δ^13^C.

The intra-sample variability is comparatively low with mean standard deviations of 0.60 and 0.69‰ for δ^18^O and δ^13^C, respectively. Six and four, respectively, samples have a standard deviation of ≥ 1‰ for δ^18^O and δ^13^C (Table 2). Four of the six samples with a high standard deviation in δ^18^O came from Florida, the other two from Mexico and Colombia. For δ^13^C highest standard deviations are shown by two Floridian sites and each one in Mexico and Brazil.

**Fig. 8:**
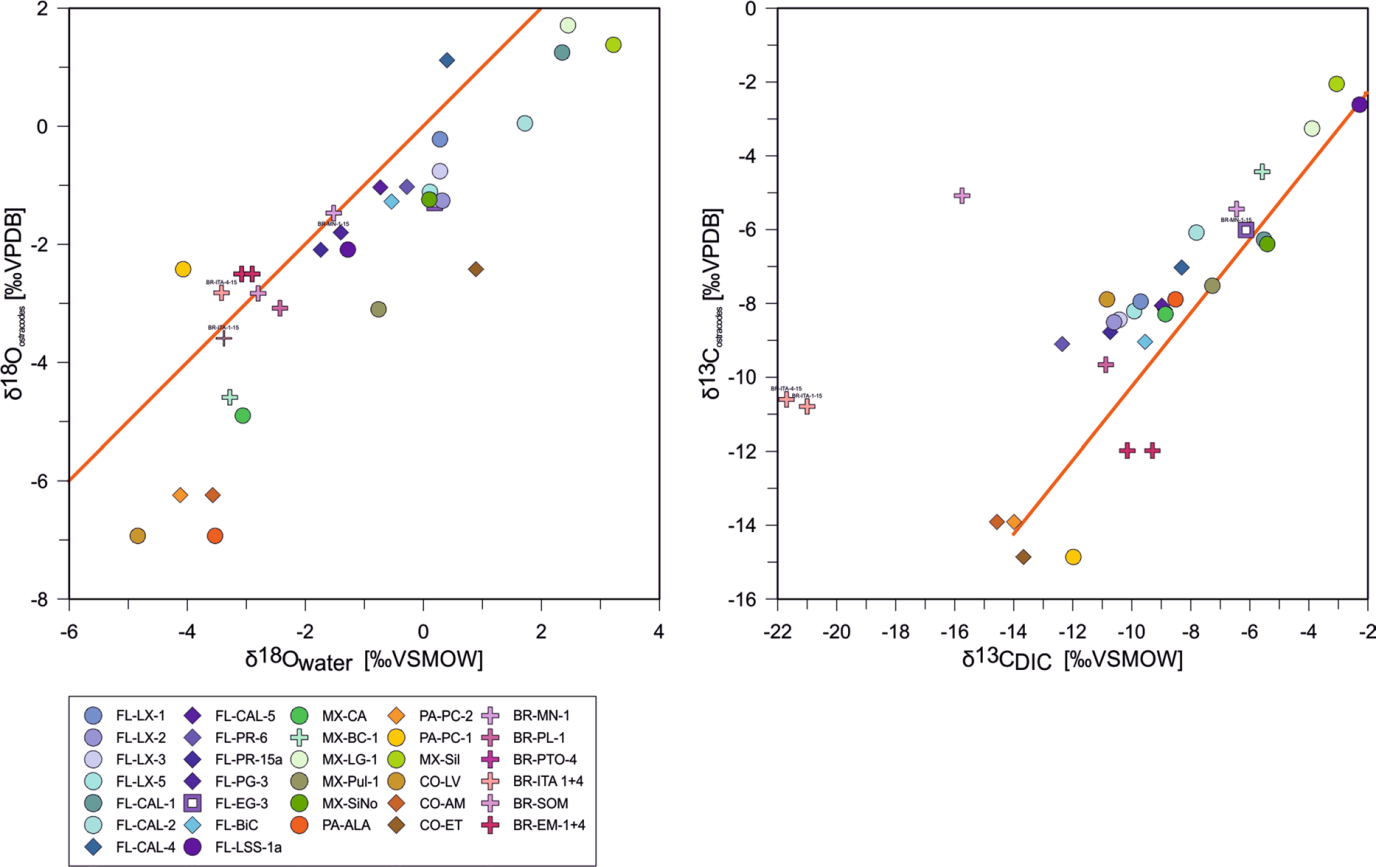
Correlations between oxygen and stable carbon isotopic composition of water (including DIC) and ostracode valves. **A** δ^18^O_water_ vs. δ^18^O_ostracode_, **B** δ^13^C_DIC_ vs. δ^13^C_ostracode_. Orange lines represent the identity lines (i.e., y = x line)

A close region-wide relationship between δ^18^O values of ostracode calcites and lake water is confirmed by high correlation coefficients of for oxygen (r^2^: 0.76), and for carbon (r^2^: 0.59), which increases to r^2^: 0.70 if the Brazilian samples with the very low δ^13^C values (BR-ITA and BR-SOM) are not considered). These high positive correlation coefficients show a strong coincidence with the line of equality (Fig. 8).

**Fig. 9:**
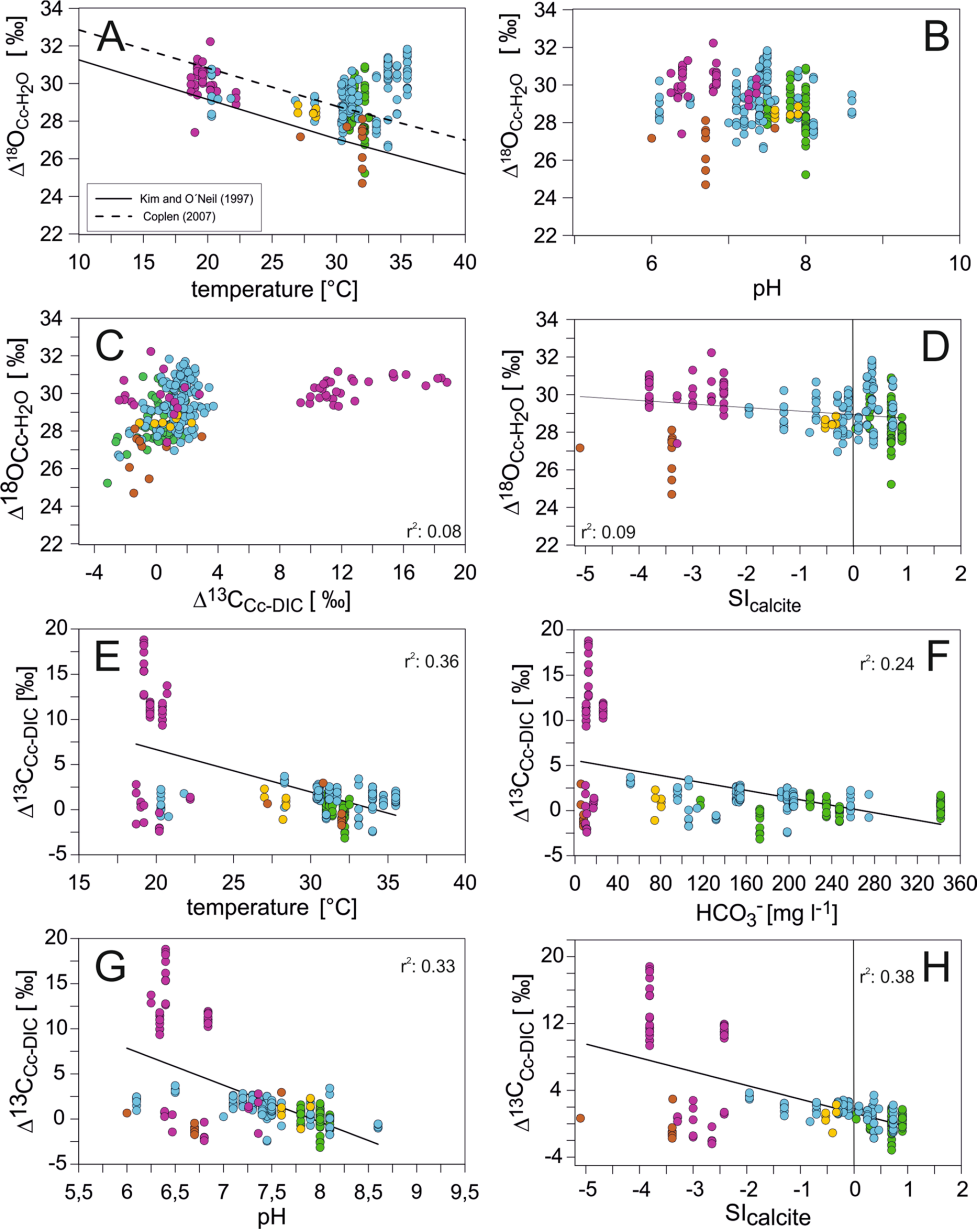
Characteristics and relationships of oxygen and stable carbon isotopes of ostracode valves. **A** Apparent oxygen isotope fractionation (1000ln(α_Cc-H2O_) ~ Δ^18^O_ostracode/calcite-H2O_ = δ^18^O_ostracode/calcite_—δ^18^O_water_) calculated from (i) measured δ^18^O_ostracode_ and δ^18^O_water_ values in comparison to (ii) oxygen isotope fractionation between calcite and water near and approaching isotopic equilibrium determined by Kim and O`Neil (1997) and Coplen (2007), respectively. **B** Apparent oxygen isotope fractionation vs. pH. **C** Apparent oxygen vs. stable carbon isotope fractionation between calcite/ostracode and water/DIC (Δ^18^O_ostracode/calcite-H2O_ = δ^13^C_ostracode/calcite_ and δ^13^C_DIC_. **D** Apparent oxygen isotope fractionation vs. calcite saturation of the solutions. Apparent oxygen isotope fractionation vs. temperature (**E**), concentration of HCO_3_^−^ (**F**), pH (**G**), and calcite saturation (**H**)

Comparison between oxygen isotope fractionation factors of the ostracodes and expected equilibrium calcites calculated by different equations show that ostracodes are enriched in ^18^O compared to the inorganic carbonate expected by Coplen (2007). Positive offsets to the calcite calculated by Coplen (2007) are displayed only by Floridian and Mexican samples associated with temperatures ≥ 30 °C. There is no correlation between oxygen isotope fractionation factors and the pH (Fig. 9A, B). According to our data, Δ^18^O seems to be unaffected by pH (Fig. 9B) and Δ^18^O values show no significant correlation with SI_calcite_ values (Fig. 9D; r^2^: 0.09). The Δ^18^O−Δ^13^C-plot shows that the majority of samples lies within a relatively narrow field of stable carbon isotope values between − 4 and 4‰ (VPDB) and oxygen values between 27 and 32‰. An exception represents some of the Brazilian samples (BR-PTO-4, BR-ITA 2-4) with very high δ^13^C values (9.3 to 18.8‰) (Fig. 9C).

The regression lines display relatively weak correlations between Δ^13^C_Cc-DIC_ and the parameters temperature, pH, and HCO_3_ with r^2^: 0.35; r^2^: 0.33; and r^2^: 0.24, respectively (Fig. 9E–G). These relationships become even uncorrelated if the Brazilian samples with the unusual high δ^13^C values are removed (r^2^: 0.07; r^2^: 0.02; and r^2^: 0.003). The strongest relationship is revealed for Δ^13^C_Cc-DIC_ and SI_calcite_ (r^2^: 0.38). Accordingly, highest Δ^13^C_Cc-DIC_ values are associated with lowest SI_calcite_ (Fig. 9H).

### Expected seasonal δ^18^O_ostracode_ range from water isotope composition

**Fig. 10:**
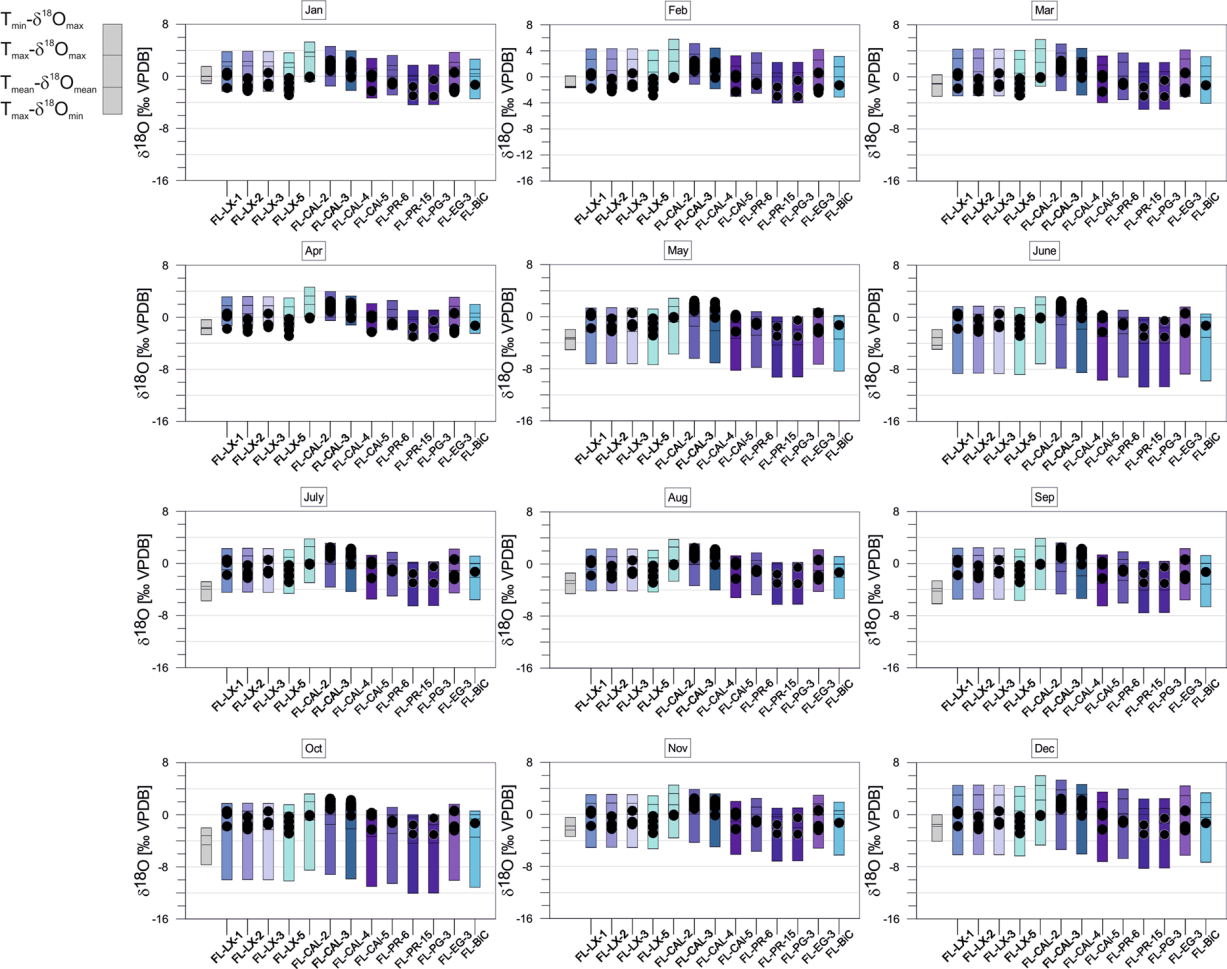
*Cytheridella* δ^18^O variations (black dots) of Floridian samples compared to the maximum δ^18^O range of calculated monthly calcite at oxygen isotopic equilibrium (bars) using air temperature and δ^18^O_water_. Grey bars refer to uncorrected calcites. Subdivision of bars is according to schematic diagram on the left side

The maximum range of variation of the expected equilibrium calcites is determined by T_min_-δ^18^O_max_ (maximum) and T_max_- δ^18^O_min_ (minimum). Mean upper (positive) values are described by T_max_- δ^18^O_max_. At almost all localities the seasonal variation of estimated equilibrium calcites reflects the variation of δ^18^O_meteoric_ (see Fig. 3). For instance, the expected equilibrium δ^18^O values of the Florida samples display largest variations during May/June and October similar to δ^18^O values of precipitation while during the beginning of the year both calcites and precipitation reveal distinctly smaller variation ranges (Fig. 10).

**Fig. 11:**
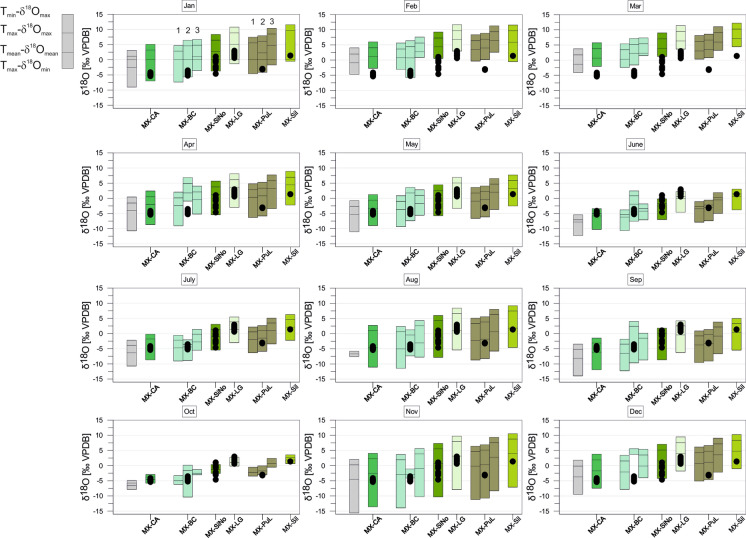
*Cytheridella* δ^18^O variations of Mexican samples compared to the maximum δ^18^O range of estimated monthly equilibrium calcites calculated from air temperatures and δ^18^O_precipiation_. Grey bars refer to uncorrected calcites. Subdivision of bars is according to schematic diagram on the left side. The numbers above the calcite ranges of MX-BC refer to different corrections with lake water (1), summer water sample value from Castro-Contreras et al. (2014) (2), and winter water sample of Castro-Contreras et al. (2014) (3). Corrections of MX-Pul were achieved with lake water (1), mean of δ^18^O value from 1990 to1995 by Curtis and Hodell (1996) (2), and a surface water value from 1993 by Curtis and Hodell (1996) (3)

The monthly variation ranges of expected equilibrium calcites from Mexican sites are largest during winter (November to April) with the most positive δ^18^O_calcites_. June and October provide not only the smallest variation ranges compared to the other months, but also relatively negative values. August is the summer month with the largest range in corrected δ^18^O_calcite_ (Fig. 11). For two localities^18^O values of water derived from the literature enabled the calculation of different corrected δ^18^O_calcites_. The resulting ranges show partly strong deviations. Especially, for MX-BC the δ^18^O_calcites_ corrected show much more negative values with our own water value than those corrected with literature data. The other locality MX-PuL shows a larger range of coincidence for all three estimated δ^18^O_calcites._

**Fig. 12:**
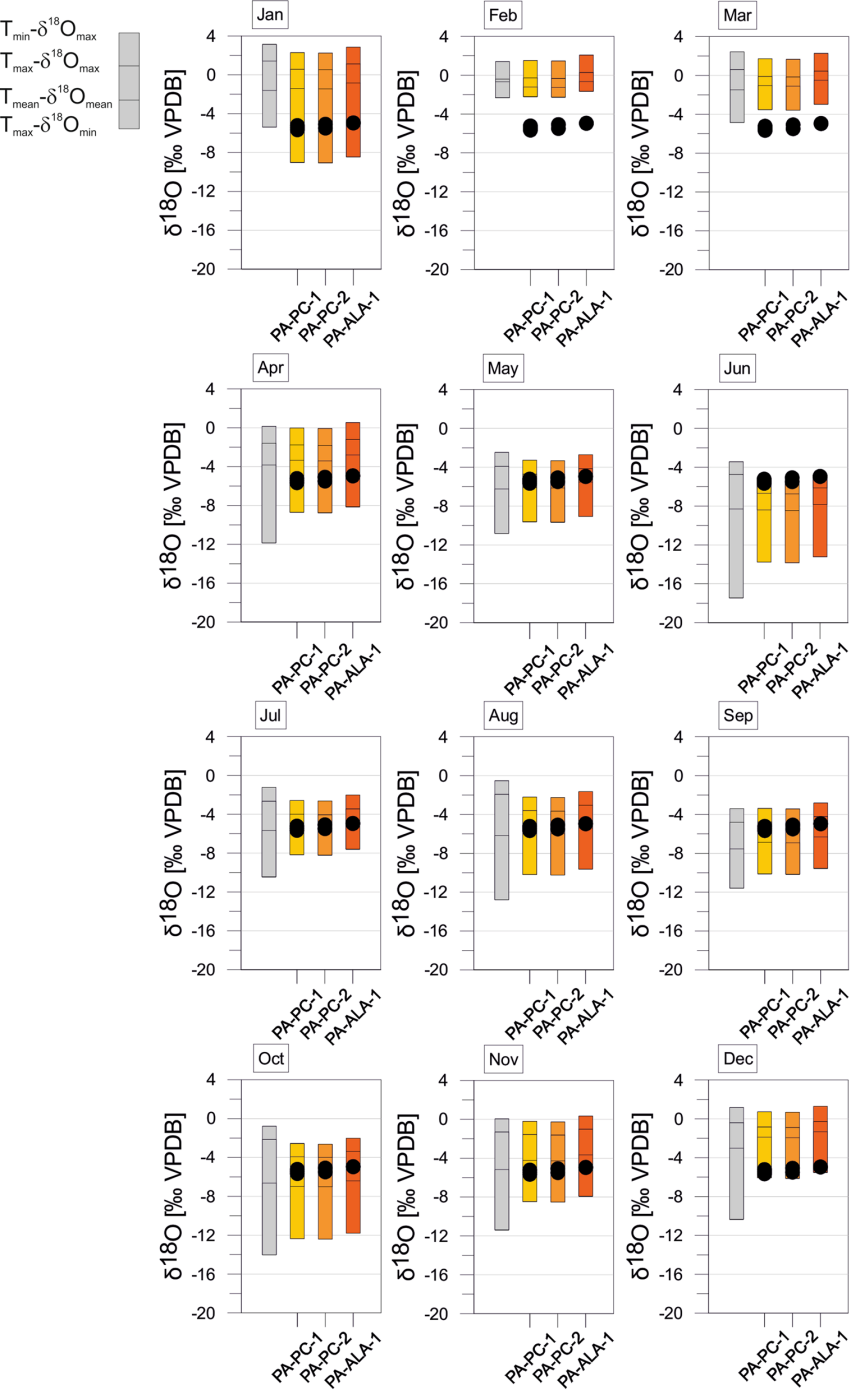
*Cytheridella* δ^18^O variations of Panamanian samples compared to the maximum δ^18^O range of estimated monthly equilibrium calcites calculated from air temperatures and δ^18^O_precipiation_. Grey bars refer to uncorrected calcites. Subdivision of bars is according to schematic diagram on the left side

**Fig. 13:**
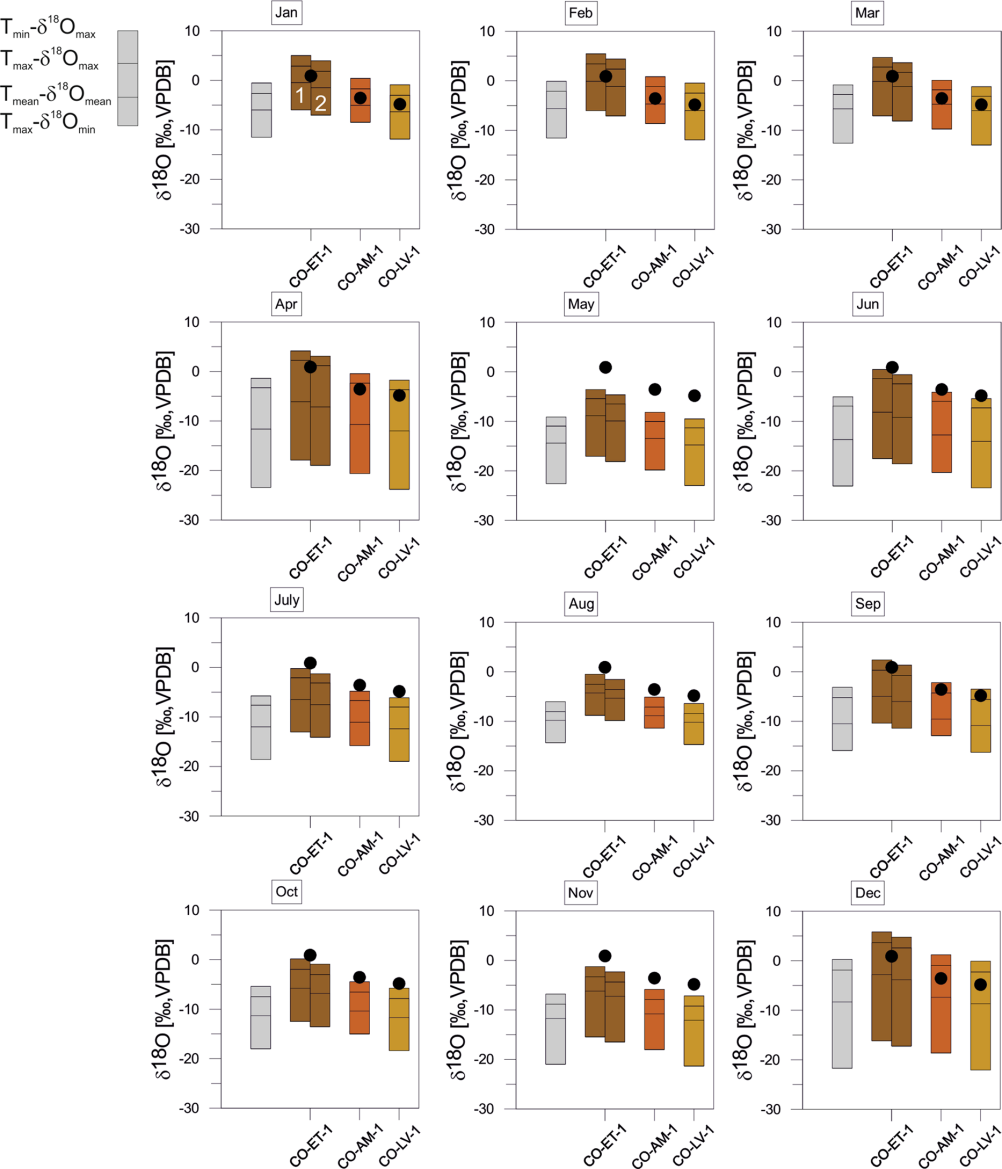
*Cytheridella* δ^18^O variations of Colombian samples compared to the maximum δ^18^O range of estimated monthly equilibrium calcites calculated from air temperatures and δ^18^O_precipiation_. Grey bars refer to uncorrected calcites. Subdivision of bars is according to schematic diagram on the left side. The two bars for the sample CO-ET refer to altitude-corrected calcite (1) and the calcite corrected with lake water (2)

**Fig. 14:**
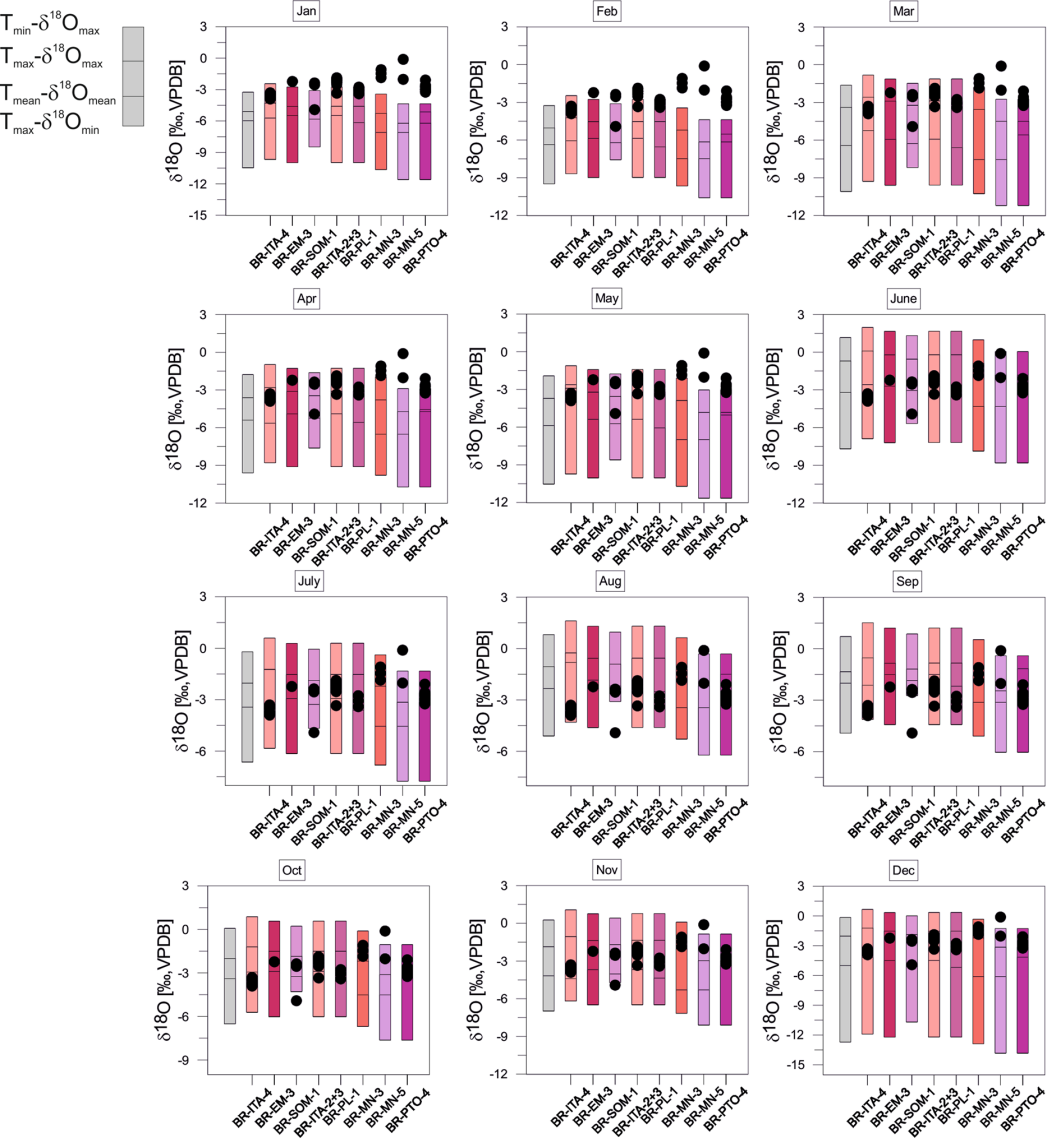
*Cytheridella* δ^18^O variations of Brazilian samples compared to the maximum δ^18^O range of estimated monthly equilibrium calcites calculated from air temperatures and δ^18^O_precipiation_. Grey bars refer to uncorrected calcites. Subdivision of bars is according to schematic diagram on the left side

The estimated equilibrium calcites of Panama provide generally large variation ranges. Largest variation ranges occur between April and October. Smallest ranges prevail in February and March. Summer months (April to November) generally provide negative values (entire variation ranges ≤ 0‰) compared to winter months during which the variation ranges become more positive (Fig. 12). Variation ranges of equilibrium calcites of Colombia vary throughout the year and provide generally negative values (≤ 0‰). Periods with relatively small variation ranges are January to March, and August and September. Large variation ranges occur from April to July, and October to December (Fig. 13). Estimated equilibrium calcites of Brazil present differing variation ranges throughout the year. The variation is solely determined by the range of negative values. Generally large ranges occur from December to May. Largest ranges and most negative values emerge in December. From June to November are the ranges relatively small with minima in August and September (Fig. 14).

The deviation between uncorrected and corrected equilibrium calcites differs between the regions. Corrected δ^18^O values of equilibrium calcites of Florida display generally much wider and more positive variation ranges than the uncorrected ones. Corrected calcites in Mexico are generally more positive than the uncorrected values. In Panama the corrected values are almost in all cases within the range of the uncorrected ones and exceed them (negatively) only in January. In Colombia the corrected calcites exhibit similar but generally less negative values than the uncorrected calcites. The corrected equilibrium calcites of Brazil differ slightly but are generally relatively similar to the uncorrected calcites. Only the two localities BR-MN-5 and BR-PTO-4 show a more negative range than the other (corrected and uncorrected) values.

### Apparent vs. expected oxygen isotope variation of ostracode valves

#### Florida

**Fig. 15:**
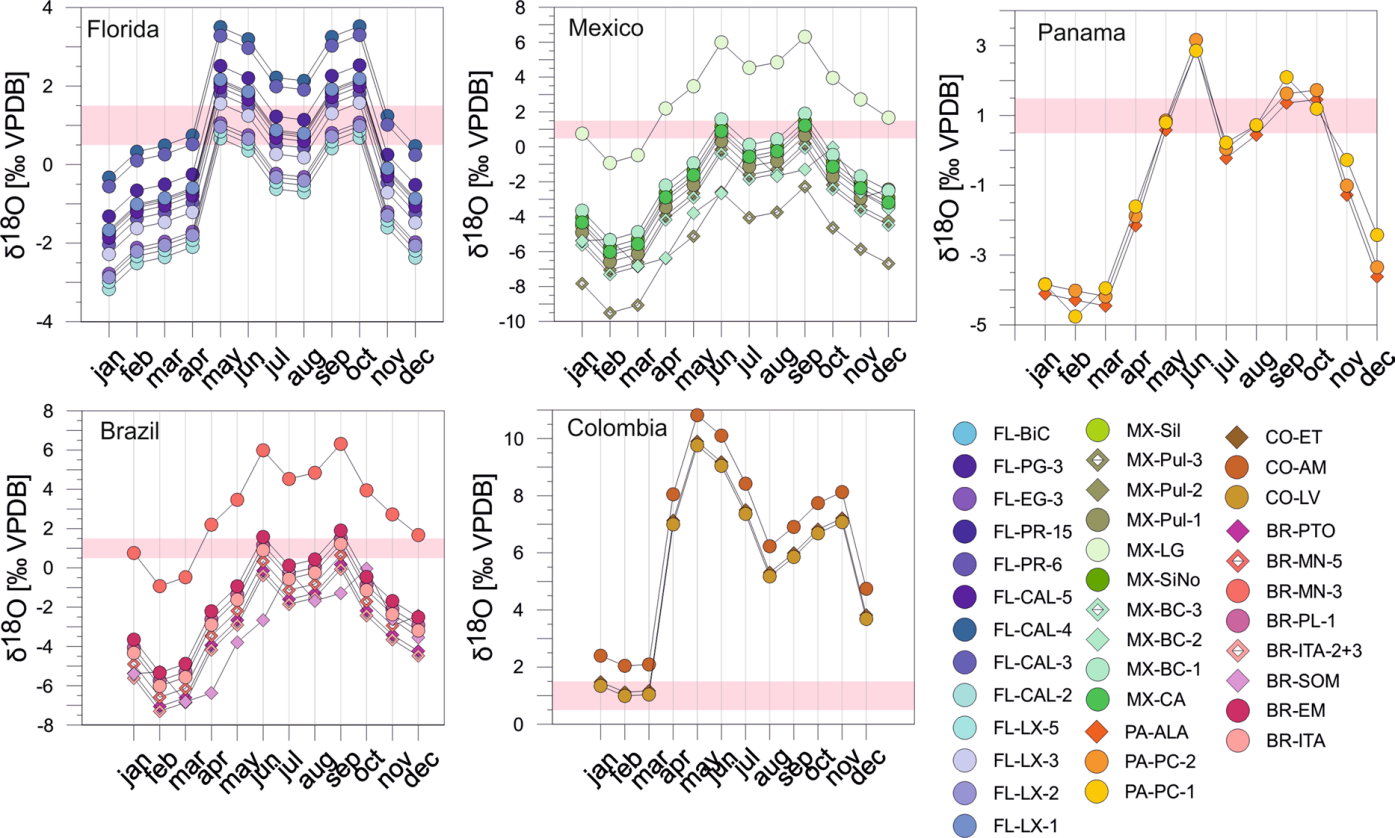
Monthly differences between mean δ^18^Oostracode and mean estimated δ^18^O_calcite_ in all regions. In each diagram is the assumed vital offset of *Cytheridella* of + 1‰ (± 0.5‰) indicated (rose bar) as reference for possible calcification periods

Generally, ostracode values vary within the ranges of the expected equilibrium calcites during winter months (November/December to March/April) and become generally more positive than equilibrium calcites during summer months (May to October). The majority of samples shows an offset of ≥ 0.5–1.5‰ between mean δ^18^O_ostracodes_ and mean δ^18^O_calcites_ during January to April and November to December (Fig. 15). Two samples (FL-CAL-4, FL-CAL-3) provide positive offsets of ~ 1‰ only in January. For all other months the offsets are far beyond the required 1‰ value. The offsets of the last group (FL-EG-3, FL-LX-2, Fl-LX-5, FL-CAL-2) match the required range of ≥  + 1‰ only during July and August. During winter (December to April) they show negative offsets to mean δ^18^O_calcites._ In November offsets are positive, but below (0 to + 0.5‰) the assumed offset of *Cytheridella* ≥  + 1‰.

#### Mexico

Ostracode δ^18^O fall below or are at the lower (negative) margin of the equilibrium calcites during winter months (December to April). A higher similarity between δ^18^O_ostracodes_ and δ^18^O_calcites_ occurs during summer months from May to November. Considering the differences between mean δ^18^O_ostracodes_ and mean δ^18^O_calcites_ (Fig. 15) it emerges that except of two samples (MX-LG, MX-Pul-3) offsets ranging between + 0.5 and + 1.5‰ are achieved in May, July, August, and October. Offsets of MX-LG are too positive and lie in the range of + 0.5 to  + 1.5‰ only during February, March, and December. Contrary, MX-Pul-3 provide only negative offsets to equilibrium calcites.

#### Panama

Ostracode δ^18^O values are within the ranges of the estimated calcites except for February and March where the δ^18^O_ostracode_ is below or at the upper margin of the calcites. Offsets between mean δ^18^O_ostracodes_ and mean δ^18^O_calcite_ correspond only during November to the required range of + 0.5 to  + 1.5‰ (Fig. 15).

#### Colombia

Ostracode δ^18^O values are within the ranges throughout the year and exhibit generally a trend to the more positive values relative to the equilibrium calcites. Offsets between mean δ^18^O_ostracodes_ and mean δ^18^O_calcite_ are far above the ~ 1‰ criterion from April to December with the strongest deviations in May and November. During January to March ostracode values fall within or are very close to the estimated calcites (Fig. 15).

#### Brazil

The ostracode values mostly coincide with the ranges of the equilibrium calcites from June to December. During the first half of the year, the ostracode values tend to be more positive relative to the estimated equilibrium calcites (Fig. 14). Differences between mean δ^18^O_ostracodes_ and mean δ^18^O_calcite_ are + 0.5 to + 1.5‰ for most localities in May, July, August, and October. Only one locality (BR-MN-3) displays offsets exceeding realistic values of up to + 1.5‰ by far (Fig. 15).

## Discussion

### Site specific hydrochemistry and ostracode formation environment

The study area provides quite heterogenous hydrochemical facies (Fig. 4; Supplementary Fig. 1) according to different background geology, and climatic and hydrological conditions. Important contributing process represents mixing of seawater and freshwater, which occurs in Florida and Brazil and to a lesser degree also in Mexico (cf. Petrini et al. 2014; Long et al. 2018).

Although saturation of calcite is affected by short-term variations (e.g., seasonal and even diurnal changes; Liu et al. 2007) the SI_calcite_ values probably roughly reflect the regional background geology differentiating the carbonate platforms of Florida and Mexico from the remaining regions which are mainly composed by silicate rocks and few or no carbonates. However, it is known that the state of the calcite saturation of a solution depends on a variety of factors including not only the calcium and bicarbonate concentrations, temperature, alkalinity, and pH but also indirectly by carbon dioxide pressure, residence time within soil and groundwater areas, as well as photosynthesis rates (Neal et al. 2002). The reason why Florida and Mexico provide differences in their SI_calcite_ values despite their similarities in lithological and climatic characteristics is attributed to striking hydrological and geochemical differences. In Florida, the chemical character of water changes systematically downgradient, owing to solution of minerals of the aquifer and corresponding increases in total dissolved solids. In Yucatán, no downgradient change exists, and dominant processes controlling the chemical character of the water are dissolution of minerals and simple mixing of the fresh water and the body of salt water that underlies the peninsula at shallow depth (Back and Hanshaw 1970). The higher amount of salt water in Yucatán provides a higher portion of dissolved solids which reduces the activity of each ion in solution and thus can dissolve more limestone than can freshwater (as in Florida) of much lower dissolved solids concentration.

High correlation coefficient (r^2^: 0.70) of SI_calcite_ and pH indicates that SI_calcite_ is largely controlled by pH (Fig. 4C). With the low SI_calcite_ values of some solutions (e.g., Fig. 4C) the question arises how ostracodes could have precipitated their valves in solutions undersaturated in calcite. It is known that calcite saturation can be highly variable with diurnal fluctuations between saturation and oversaturation in rivers (Neal et al. 2002). Also, changes from undersaturated to saturated conditions are reported on long-term (decades) and seasonal time scales (Ulloa-Cedamanos et al. 2020). It cannot be ruled out, however, that the water bodies may have changed from undersaturation to saturation within weeks—the time frame assumed between the precipitation of the valves and sampling. Actually, biomineralization by ostracodes is not very well understood. It is known that prior to molting the ostracode accumulates huge amounts of carbonatic and phosphate granules in the outer epidermal cells which form calcitic crystals via an intermediate step. Therefore, it is speculated that ostracodes may store calcium internally what contrasts to the common assumption that the calcium secreted by the animal originates directly from the ambient water (Keatings et al. 2002; Keyser and Walter 2004). This was, however, rejected since carbon isotope fractionation is not similarly affected by vital effects as oxygen isotopes (Keatings et al. 2002).

Lakes exhibit a broad range of δ^13^C_DIC_ values, and the variation between lakes is generally larger than seasonal variations within a lake (Bade et al. 2004). It is assumed that biological productivity within a lake is a major control on δ^13^C_DIC_ signatures (e.g., Leng and Marshall 2004). However, additional geochemical factors such as the dissolution of limestone in karst areas or different pathways of plants for fixing CO_2_ (i.e., C3 and C4 plants), or more specifically, their interaction with lake metabolism contribute largely to the δ^13^C_DIC_ signature (Bade et al. 2004). Although our dataset comprises samples from a variety of water bodies of different sizes, hydrological and productivity characteristics. Our analyses displayed a relatively strong (positive) relationship between pH and δ^13^C_DIC._ This indicates that fractionation between CO_2_ and carbonate species at different pH values contributes a certain amount to the variation of δ^13^C_DIC_ in the solutions. Through carbonate chemistry, the correlation between pH and δ^13^C_DIC_ is probably not independent from the pattern observed with SI_calcite_. This implies that the regional geology (i.e., calcareous vs. siliceous dominance) is responsible for the major picture. However, DIC in aquatic ecosystems is maintained by several mechanisms that include, but are not limited to, dissolution of atmospheric CO_2_, terrestrial runoff, stream, and groundwater inputs, and oxidation of organic matter from the water column and sediments that all can have locally very different influence. Lacustrine systems often receive DIC inputs from more than one source, and the resulting isotope ratios typically reflect the `weighted average´ of these sources (Gu et al. 2004). Therefore, local conditions probably represent a stronger control on δ^13^C_DIC_ in the solutions than the overall variation in pH and SI_calcite_.

It has been observed that in carbonate springs calcite saturation leads to significant δ^13^C_DIC_ increase (Abongwa and Atekwana 2015) due to CO_2_ degassing which preferentially releases ^12^CO_2_ (Liu et al. 2003). Concluding from SI_calcite_ ≥ 0 this effect can be assumed to a part of the solutions. Lake δ^13^C_DIC_ variations differ widely in carbonate and carbonate-deficient (or hard and soft water) systems. In carbonate systems the seasonal fluctuations of δ^13^C_DIC_ display more positive values and distinctly lower amplitudes with max. ~ 6‰ (Myrbo and Shapley 2006) compared to soft water lakes in which seasonal δ^13^C_DIC_ values can span a wide range of ~ 10‰ (Herczeg 1987). Nonetheless, our data probably show no consistent picture or, more specifically, are hard to interpret due to large differences in geological and hydrological conditions, and restricted number of measurements.

In hydrologically closed water bodies, the correlation between [Mg/Ca] and salinity (i.e., conductivity) arises as a result of CaCO_3_ mineral precipitation due to the differences in mineral saturation, which removes Ca^2+^ from the lake water and leads to an increase of the [Mg/Ca] ratio (Fukushi and Matsumiya 2018). The independence of Mg/Ca and conductivity of the solutions highlights thus the different and rather open hydrologies of the investigated water bodies. Also, many localities are situated close to the sea and provide strong differences in the geological background. Thus, the varying combinations of freshwater mixing with sea water and/or the influence of groundwater draining, e.g., dolomitic rocks probably control the differences of [Mg/Ca] in the study area (e.g., Surge and Lohmann 2002).

### Parameters controlling the isotopic variability of calcite valves of *Cytheridella*

The oxygen isotope composition of ostracod valves is a function of the temperature and the isotopic composition of the lake water in which the biominerals were secreted, modified by a vital offset, a systematic, species-specific deviation from the ^18^O value of a theoretical calcite precipitated under equilibrium conditions (von Grafenstein et al. 1999; Decrouy et al. 2011). The oxygen isotopic composition of the lake water is determined by the atmospheric component of the global hydrological cycle (e.g., Rozanski et al. 1993). Lake waters reflect the mean oxygen isotopic composition of catchment precipitation, which is primarily a function of latitude, modified by orography and continentality (Schwalb 2003). Variations in δ^18^O of precipitation are mainly determined by temperature in middle and high latitudes (Jouzel et al. 2013), while precipitation amount is the main determining factor in the tropics (Lachniet and Patterson 2006, 2009). The combination of (precipitation) amount and temperature effects is common in subtropical latitudes (Bowen 2008). The isotopic composition of lake water depends on the isotopic composition of the precipitation in the catchment (as described above), and the processes that affect the isotopic composition in the lake such as evaporation that changes with relative humidity, temperature, wind stress, relation of lake area vs. volume, and residence time (Schwalb 2003).

With a few exceptions, ostracode δ^18^O/δ^13^C correspond very well to water δ^18^O/ δ^13^C_DIC_ (Fig. 8). This highlights the potential of ostracodes as reliable proxies for water chemistry, as shown by previous studies (e.g., von Grafenstein et al. 1999; Schwalb 2003; Marco-Barba et al. 2012; Pérez et al. 2013). Marco-Barba et al. (2012) observed that ostracode oxygen fractionation decreases with pH explained by the shrinking pool of HCO_3_^−^ which is used by ostracodes for calcification. Although our dataset also comprises a pH range (5.3 to 8.6) in which HCO_3_^−^ is the dominant inorganic carbon species, our data do not indicate that values of equilibrium calcites have changed in response to changes of pH. The differences between lake water δ^13^C_DIC_ and δ^13^C_ostracode_ (i.e., Δ^13^C) exceed known fractionation factors at 25 °C that lie in the range of 0.35 to 2.3‰ (Mucci and Morse 1990) and between 0.9 and 1 (Romanek et al. 1992) by far. While Marco-Barba et al. (2012) found generally negative offsets in the range of 0 to − 4‰ our data show no systematics. Especially, removing the Brazilian samples with the unusual δ^13^C values leads to uncorrelated relationships with temperature, pH, and SI_calcite_ (Fig. 6E–H). One reason for this might be attributed, again, to the temporal lag between calcification and sampling that leads to comparison with a `wrong´ δ^13^C_DIC_ value. Observed negative offsets are suggested to be caused by infaunal molting. Within the sediment is water DIC strongly affected by release of CO_2_ enriched in ^12^C due to remineralization of organic matter, which may also have lower δ^13^C values and could be incorporated in the δ^13^C values of species burrowed in the sediment (Decrouy et al. 2011; Marco-Barba et al. 2012). Since most of the values are positive this cannot be attributed to *Cytheridella*.

The majority of oxygen values of *Cytheridella* valves display relatively small ranges (~ 2‰) which is typical for small to small-medium open lakes according to the conceptual model by Leng and Marshall (2004). Larger ranges in the δ^18^O values indicate either seasonal changes or closed basin lakes. Sites with flowing conditions such as the Floridian river sites may therefore reflect seasonal changes. This is supported by measurements on *Cyprideis* valves sampled in December and July in the same sites which show a similar range in oxygen isotopes (Meyer et al. 2017b).

The largest range of > 5‰ is displayed by a Mexican cenote (MX-SiNo). Cenotes can be lotic and lentic and are mainly fed by groundwater (Schmitter-Soto et al. 2002). In closed-basin tropical to subtropical lakes with a seasonally dry climate, the δ^18^O of lake water is controlled mainly by the ratio of evaporation to precipitation (Curtis and Hodell 1996 and cited references therein). The relatively large difference in oxygen isotope values of groundwater and rainwater (− 3.91‰, Curtis and Hodell 1996; − 4 to − 5‰, Wassenaar et al. 2009) to the lake water (0.10‰, Table 2) indicates that the cenote MX-SiNo loses a significant fraction of its hydrological budget to evaporation.

Groundwaters and river waters, in general, have typically low δ^13^C_DIC_ values between − 10‰ and − 15‰ (VPDB; Leng and Marshall 2004). Higher δ^13^C_DIC_ (around − 8‰ to − 12‰ VPDB) in groundwaters may occur in karstic regions where dissolution of catchment limestones is more pronounced than CO_2_ uptake (e.g., Emblanch et al. 2003; Marfia et al. 2004; Han et al. 2010). In hydrologically closed lakes, carbonates often display covariance of δ^13^C and δ^18^O values likely reflecting different degrees of equilibration with atmospheric CO_2_ and preferential evaporative loss of the ^16^O (Leng and Marshall 2004). In our dataset there are two localities in Yucatán (MX-LG, MX-Sil) showing both high δ^13^C and δ^18^O probably indicating a long exposure of the lake water to surface and exchange with the atmosphere. As discussed in Meyer et al. (2017b), δ^13^C clearly differs between marsh and river sites which is interpreted as result from high biological activity and differences in the residence time of the water. Photosynthetic activity will decrease ^12^C from the water, due to the preferential uptake by aquatic plants, while respiration has the opposite effect (e.g., Leng and Marshall 2004). Marshes are characterized by low water levels, stagnant water, and dense aquatic vegetation. Residence time of the water in those systems is long (Childers 2006) enabling the accumulation and consumption of organic matter which will contribute to increases of δ^13^C. In rivers and canals, large-scale processes such as the input and mixing of inorganic carbon from different sources (groundwater, tributaries, etc.) in the catchment is more important than local small-scale processes. Differences in δ^13^C_DIC_ between different rivers within a region reflect different residence times of water in the tributaries (Atekwana and Krishnamurthy 1998a, b).

Lowest δ^13^C values from Colombian sites (CO-ET, CO-AM) correspond well with the Amazon River in the transition between Andean upland and lowland (− 14.5‰ ± 1.7; Quay et al. 1992). The geology of Colombia provides virtually no carbonates (cf. Parra et al. 2009). Streams draining silicate rocks have low δ^13^C_DIC_ values possibly due to their relatively low pH values resulting in a relatively high content in dissolved CO_2_ vs. bicarbonate ions (Hélie et al. 2002). Comparable conditions prevail in Panama contributing to relatively low δ^13^C values. The low values may be due to enhanced supplies of ^13^C-depleted DIC from soils and groundwaters from watersheds and/or high oxidation rates of dissolved or particulate ^13^C-depleted organic carbon (cf. Hélie et al. 2002; Leng and Marshall 2004).

The very low δ^13^C values of some Brazilian sites correspond to groundwater sites in which no dissolution of carbonates takes place (Sracek and Hirata 2002). Although variation with values around − 16 to − 21‰ is considerably, this does not provide hints of the much more positive ostracode values from one site in this water body. The most logical explanation for this phenomenon is the local mixing with ^13^C enriched sources that may vary through time explaining the relatively large range at the site. Pedrozo and Rocha (2007) report a gradient in nutrient and ion concentration, conductivity and other parameters, even within lakes, reflecting different sewage inputs that might be also the case for Lagoa Itapeva. The process leading to the distinctly higher δ^13^C values could include bacterial methanogenesis that leads to a strong ^13^C fractionation between CH_4_ and CO_2_, because the metabolic pathway of methanogenic bacteria favors the light isotopes. Carbonates formed under this condition have markedly positive δ^13^C values (Talbot and Kelts 1990; Schwalb 2003). The question is if this may occur spatially and temporally restricted in a water body as indicated here.

The within sample variability of ostracodes varies between all sites. Sites with a relatively high within-sample variability (standard deviation ≥ 1‰) occur mostly in Florida. For living populations, some variability can undoubtedly be attributed to temporal fluctuations in water temperature or composition during the ostracode´s life, or small-scale spatial differences, which will mean that the individual valves did not calcify under truly uniform conditions (Holmes 2008). Contrary to the other regions, most of the sample localities in Florida are rivers or channels whose isotopic composition is usually primarily controlled by precipitation (e.g., Henderson and Shuman 2010; Price et al. 2008). Temporal and spatial fluctuations in river water can result from tributary/lake water mixing, damming regulation and temperature (Wu et al. 2018). In lakes, precipitation water is mixed with a great volume of older evaporated water, buffering the δ^18^O variations of the water (Leng and Marshall 2004), and can explain the lower variation of δ^18^O_ostracodes_ in lakes. An additional contribution to the within-samples variability derives from the life cycle of the ostracodes. Although we used only specimens which were alive at time of sampling ensure that the calcification time was closed to the time of sampling, it cannot be excluded that the adult life span of *Cytheridella* is long enough to assemble (living) specimens from more than one molting periods in a sample. Generally, it has been shown that a number of ~ 10 ostracode valves from a stratigraphic interval are needed to remove the influence of high-frequency environmental variations (Xia et al. 1997; Dixit et al. 2015). Escobar et al. (2010) estimated that this number varies and can be higher in dependence of hydrological conditions (i.e., lake level fluctuations in littoral areas), and inter-annual climate variability of the study area. The weak positive relationship between sample size and oxygen isotope range (Fig. 6C) is caused by single localities and does not display a coherent picture for the whole dataset or an entire region. This implies that the increase of intra-sample isotopic ranges with samples size is caused by local differences in the hydrological conditions (i.e., lake morphology and resulting sensitivity for water balance changes) and does not display a general relationship.

As expected, the apparent oxygen isotope fractionation of ostracode—H_2_O shows a clear correlation with temperature with lesser fractionation at higher temperatures (Fig. 9A). This is in agreement with previous studies which observed that ostracode oxygen isotopes fractionation increases with temperature (e.g., Xia et al. 1997; Li and Liu 2010). Interestingly, at temperatures between 30 and 37 °C fractionation exaggerates equilibrium much stronger than the anticipated vital effect of ~ + 1‰. This could be due to a kinetic effect explained by an amorphous precursor pathway of the precipitated ostracode calcite (cf. Dietzel et al. 2020). However, although ostracodes were not as intensively studied for their biomineralization as other organism groups it is known that the valves of Cytheroidea, to which *Cytheridella* belongs, are almost completely built of calcite crystals (Keyser and Walter 2004).

As stated previously it is more probable that the sample solution and measured temperature do not correspond to those at the time of valve calcification. Rapid valve calcification and strong variability of some environmental parameters preclude to correlate values representing exactly the same specific time period. The `unusual´ high fractionations within the temperature range of 30 °C to 37 °C affects almost exclusively samples from Florida and Mexico. Sampling took place in summer where daily temperature gradients in South Florida account ~ 2 °C and increase up to 4–5 °C between months (e.g., Price and Overton 2005).

### Accuracy of expected equilibrium calcites

The accuracy of the expected equilibrium calcites depends on a variety of parameters.

Water temperature data of the study area are only available for Florida and for some localities in Yucatán. Due to the strong relationship between mean air temperature and mean water temperature (r^2^: 0.80; Rollinson and Rowe 2018) we used air temperature data for the calculation of expected equilibrium calcites. However, this relationship weakens from high to low latitude lakes probably due to the lower inter-annual air temperature variability in the tropics. Additional influences represent geomorphic factors such as lake surface area and lake depth (Kraemer et al. 2015).

In cenotes, water temperature is fairly constant within the year with a gradient of ~ 2–3 °C, and seasonal water temperature differences are, thus, negligible (Alcocer et al. 1998). If this is true, our field measurements of 31–32 °C indicate that the minimum (air) temperatures used for the calculation of the equilibrium calcites are much too low (Table 1; Fig. 2). Indeed, minimum water temperatures of 24.7 or 24.8 °C, respectively, (Alcocer et al. 1998; Pérez et al. 2010) imply that inclusion of minimum air temperatures is not necessary.

Temperature seasonality is much higher in marginal regions of the study area (Florida, S-Brazil). Temperature time series of Caloosahatchee River demonstrate that lowest mean water temperature occurs in January with 18.7 °C and highest mean water in July with 30.4 °C (Baldwin and Hunt, 2014). Mean air temperatures of nearby Fort Myers range from 17.4 °C in January to 28.1 °C in August (climate-data.org). Thus, the water temperatures are slightly higher but correspond very well to the air temperatures. Similar conditions can be assumed for Southern Brazil. A large lake ~ 2–3° south to the sample area in S-Brazil displays a seasonal temperature range of 8–25 °C (Tavares et al. 2019). The range is slightly higher reported for several rivers and lakes in the state of Rio Grande do Sul with 9–28 °C, respectively (Garcia et al. 2008). The deviation to the seasonal air temperature range of Porto Alegre with 10.3–29.6 °C is relatively small. This implies that the temperature range used for the calculation of the equilibrium calcites represents realistic estimates of lake water temperatures.

Another fundamental assumption for the estimation of the equilibrium calcites is that δ^18^O_precipitation_ represents the major control on lake water δ^18^O. Spatial variability of δ^18^O (and δD) composition of precipitation are negatively correlated with temperature, latitude, altitude, distance from the coast, and the amount of precipitation. Further overriding factors that influence the isotopic signature of local precipitation are the continual loss of moisture from an air mass as it moves away from its evaporation source and mixing of different air masses from local vapor sources as well as storm trajectory (Price et al. 2008 and references therein). Therefore, the position or more specifically the distance of the GNIP stations to the sample localities is an important point. GNIP stations are unevenly distributed throughout the study area and are relatively far away from the sample localities in cases of Yucatán and Colombia which questions the representativeness of the δ^18^O precipitation data for the equilibrium calcites.

The air-line distance from Veracruz to Central Yucatán is over 700 km. However, Veracruz and at least the southwestern parts of the Yucatán Peninsula provide similar climatic conditions and annual precipitation amounts of 1200–1500 mm. Isoscape maps of shallow groundwater, that is assumed as proxy for integrating long-term (ca. 5–10 years.) precipitation infiltration inputs, show that Veracruz provides values generally ~ 1‰ lower compared to Yucatán (Wassenaar et al. 2009). Thus, the equilibrium calcites for Yucatán could have been estimated too low.

Although air-line distance between Bogota and the sample localities is only between 100 and 230 km there is, however, an altitude difference of ~ 2170 m (difference refers to Villavicencio). In order to test for this effect, we compared the correction with lake water sample and the altitude (Fig. 13). The deviation between the altitude-corrected and the lake water-corrected calcite is negligible small. This indicates that the correction with local water samples is sufficient to compensate the difference in altitude.

The seasonal fluctuations of precipitation amount and its isotopic composition over the major part of South and Central America are controlled by seasonal displacement of the Intertropical Convergence Zone (ITCZ) and the associated changes in the circulation patterns and moisture transport across the continent (Rozanski and Araguás-Araguás 1995). In the tropical Americas δ^18^O_precipitation_ is not only related to one climatic variable but is rather assumed to result from the interplay of different factors which include precipitation amount, temperature, source region contribution, and also the atmospheric circulation (Vuille et al. 2003). For these reasons the inclusion of more than one water sample per site that illustrate seasonal changes of the lake water would be beneficial for the correction of the equilibrium calcites.

Meteoric water lines of surface water in high-humidity regions such as Panama imply that they are not affected by substantial evaporation (Lachniet and Patterson 2006). Our data which were taken during the rainy season show the same subordinate influence of evaporation on δ^18^O and δD values of the sample sites (Fig. 5A). Corrections of the calcites that are primarily intended to provide an approximation of the evaporation effect might be therefore not as similarly necessary as in less humid regions and closed basin lakes. However, since all regions except Southern Brazil provide a pronounced precipitation seasonality the necessity of corrections probably differs throughout the year.

The inclusion of literature data to receive further equilibrium calcites shows that the ostracode values coincide stronger with the lake water-corrected calcites than with the literature data-corrected calcites (e.g., Fig. 10). This implies that the lake water has not changed its isotopic composition strongly during calcification of the ostracode valves and (lake water) sampling. The usage of lake water compositions from different dates (i.e., years) might be useful for estimations of different lake conditions for application to, e.g., fossil ostracode valves.

### Inferences on *Cytheridella* calcification periods

To identify possible calcification periods for *Cytheridella* we compared estimated monthly ranges of equilibrium calcites with the ostracode δ^18^O values (Figs. 10, 11, 12, 13 and 14). Meyer et al. (2017b) excluded months with a large δ^18^O_eq_ex_ range as calcification period and assumed that for a plausible calcification time, the ostracode value lies within the range of the theoretical calcite. Further, δ^18^O_ostracode_ should tend to be more positive due to positive vital effect. These assumptions are, however, imprecise in the way that it is not clear which δ^18^O_eq_ value should be exceeded by the δ^18^O_ostracode_; for instance, the value calculated on the basis of T_mean_ and mean δ^18^O_meteoric_, the mean of the calcite range or the upper range margin of the calcite (representing T_min_ and δ^18^O_max_). Alternatively, the requirement that the values should tend to be more positive could also indicate that δ^18^O_ostracode_ is just over the calcite range. This precludes unequivocal identification of possible calcification periods. The approach using the differences between mean δ^18^O_ostracode_ and the equilibrium calcite value based on T_mean_- δ^18^O_mean_ facilitates the recognition of possible calcification periods.

The most conspicuous finding of this approach is the conformative pattern of offsets between mean equilibrium calcites and mean ostracode isotope values during the year (Fig. 15). Although it is possible that through different factors (see discussion above) the equilibrium calcites may have under- or overestimated (displayed by large differences in the offsets) it becomes apparent that there is a general pattern displayed by all regions in form of an `M´. This pattern indicates that winter months can be excluded since offsets are distinctly negative. An exception is provided by Floridian offsets that are relatively positive compared to other regions. This might be attributed to the relatively small ranges of the equilibrium calcites during winter (Fig. 10). Some summer months (in most regions June and September) can be also excluded due to offsets far too positive.

Although each region displays different offsets throughout the year there are periods or months in which almost all offsets coincide with the ~ 1‰ (± 0.5‰) boundary representing the assumed vital effect of *Cytheridella* (see Fig. 15). Accepting the 1‰-boundary as valid specific offset of *Cytheridella*, two or three possible calcification periods in spring (April/May), mid-year (July and August) and autumn (October/November) are indicated. This coincides with the observation by Pérez et al. (2011) who reported that *Cytheridella* molts in spring. It is important to consider that the diagram displays only in which months the offsets are in the suitable range of about + 1‰. Meyer et al. (2017b) assumed also that *Cytheridella* has a possible calcification period in spring (April) with a second possible calcification time in autumn (October).

The within-sample isotopic variation can provide indirect hints on the life cycle of the ostracode species. The relatively low variation ranges of most sites indicate short and probably seasonally restricted calcification periods of the respective population. Unfortunately, little is known about total life spans in general, and adult life times in particular, of most ostracodes. Within the Podocopida, to which all non-marine ostracodes belong, the Cytheroidea are assumed to exhibit total life spans of ~ 2 months to > 3 years, and adult life times of > 7 days to 3 months (Cohen and Morin 1990). From *Metacypris cordata*, a member of the Subfamiliy Timiriaseviinae to which *Cytheridella* is assigned, is reported to have a total life span of 6–10 months with one generation (Colin and Danielopol 1979).

So far, our approach ignores variations in δ^13^C. However, since processes determining carbon isotope values differ almost completely from that controlling oxygen (e.g., Schwalb 2003; Leng and Marshall 2004) it might be possible to improve our understanding on the relationships between seasonal variations on carbon δ^13^C_DIC_ and its imprint in authigenic carbonates such as ostracodes’ valves.

In littoral zones the δ^13^C_DIC_ experiences large seasonal variations (Decrouy et al. 2011). The knowledge of these variations could eventually help to determine if *Cytheridella* develops a second generation in cases when the oxygen isotopes provide inconclusive information. So far, investigations of the morphological variability (Wrozyna et al. 2018a, b, 2019) did not give any hints of e.g., seasonal morphotypes. Ultimately, the population age structure of living *Cytheridella* samples from different months or seasons would provide the necessary proof of a second generation.

Nonetheless, the similarity of the pattern implies that the calcification time (within a year) of *Cytheridella* is similar in all regions. The synchronous life cycle of *Cytheridella* from the different regions is somewhat surprising, since life histories or even development phases of crustaceans in general, are often adapted and coupled to marked shifts in their environment (Olesen 2018). For ostracodes it is reported that speed of development has been found to be related to environmental factors, particularly temperature, salinity, and habitat (Cohen and Morin 1990 and references therein). If this would be the case for *Cytheridella,* another pattern would emerge since our data set covers a wide latitudinal range, different habitats, hydrochemical facies and salinity ranges (within and between the regions). In contrast to temperate regions where seasonality is probably controlled by temperature (Horne 1983) it has been hypothesized that seasonality of tropical ostracodes might be related to hydrological conditions (i.e., precipitation). As a consequence, the calcification periods of *Cytheridella* are therefore to be related to the shift between the dry and rainy season (Meyer et al. 2017b). However, according to our data this hypothesis has to be rejected since there are pronounced differences in annual precipitation distribution and amounts between the studied regions (Fig. 2). In particular, Southern Brazil is characterized by precipitation throughout the year compared to the other regions where precipitation is restricted to summer months.

As an alternative view we can consider that a given life cycle is also the result of its ancestry, meaning that it is best interpreted by implementing not only ecological but also evolutionary interpretations (Olesen 2018). Thus, phylogenetical relationships are highly relevant to understand shared similarities of life cycles while ecological studies could identify short term and local explanations for differences in certain developmental phases due to environmental factors such as food availability, hydrological changes, etc. Morphometric investigations of appendages and valves have shown that *Cytheridella* develops distinct regional morphotypes (Wrozyna et al. 2016, 2018a). Correspondingly, it has been shown that freshwater ostracodes in the Neotropical realm are characterized by a high proportion of endemism (Cohuo et al. 2017). More recent speciation(s), which are usually not detected in qualitative studies (Wrozyna et al. 2019), could explain why morphological divergence is not recognizable. Therefore, the life cycle of *Cytheridella* seems to be phylogenetically inherited and linked to a supraspecific level. It might have originally been adapted to environmental conditions but has been conserved during the migration and radiation of the group over the Neotropical realm. To support this hypothesis, it must be tested if other taxonomical groups (e.g., species, genera, families) exhibit the same or a similar pattern in order to identify the role of phylogenetic relationships and environmental influences.

This new approach enables a better insight into calcification conditions and resulting geochemical (i.e., δ^18^O, δ^13^C) signatures of ostracode valves on a seasonal basis in (sub-)tropical regions where data on lake water hydrochemistry are mostly missing. It also contributes to biological inferences as reconstructed calcification periods of the ostracode species provide hints on its life cycle. Future studies should include monitoring of important hydrochemical parameters (pH, water temperature, δ^18^O and δ^13^C_DIC_, etc.) in order to improve the understanding of temporal fluctuations and interrelationships of involved components on the calcification processes, and repeated sampling of ostracode specimens and also the water in which they live which would help to verify findings of the `calculated´ calcification periods.

## Conclusions

For the first time this study investigates the relationships between physicochemical/environmental conditions and the isotopic composition of calcitic valves of recent populations of the common Neotropical ostracode *Cytheridella* over a large geographical range. We extended a newly developed approach based on the estimation of δ^18^O values of monthly expected equilibrium calcites as references for the interpretation of ostracode δ^18^O.

Generally, δ^18^O values of ostracode and water correspond very well. Despite limited knowledge on the temperature constraints of the samples localities it is indicated that the expected oxygen isotope fractionation of ostracode—H_2_O correlate with temperature displaying to smaller fractionation at higher temperatures. Exceptions, such as unusual high offsets of δ^18^O values from isotopical equilibria probably reflect the time lag between valve calcification and sampling. Since our dataset includes a wide range of water bodies and the inferences on ostracode calcification periods are similar, it can be assumed that this approach is applicable not only to flowing water but also to (open) lakes. As postulated in other studies, δ^18^O precipitation and temperature are the most important controls on lake water and, consequently, in ostracode δ^18^O valves. The overall plausibility of the data proves that the use of lake water analyses as approximation for an evaporation effect on the δ^18^O of precipitation values used for the calculation of equilibrium calcites is sufficient, at least for the sampling season. Corrections of the monthly estimated equilibrium calcites were done by single lake water samples. Uncertainties about high-frequency or seasonal variations of local water isotopes composition caused by mixing, evaporation, precipitation, hydrological conditions, etc. may lead to over- or underestimation of equilibrium calcites or isotope fractionation which ultimately challenges the application of ostracodes for long-term means of δ^18^O. On a different point of view, ostracode carbonates record even those subtle high-resolution variation in water composition due to rapid precipitation of their valves. For accurate estimation of expected calcites at isotopically equilibrium, the temperature and/or solution composition at the time of calcification must be known or, more preferable, need to be available within a time series data set at least at seasonal resolution. Future studies should therefore be based on monitoring data sets of water temperature and hydrochemistry (pH, chemical composition, δ^18^O, δ^13^C_DIC_), and autecological characterization (i.e., life cycle) of the ostracode species.

The region wide comparison of δ^13^C values shows that important parameters represent the hydrology in terms of residence time and the geology in the catchment area. However, with few exceptions δ ^13^C_ostracodes_ are almost identical to δ ^13^C_DIC_, thus can be used as proxy of local origin and variability of carbon source for ostracod valve formation.

Inferences can be drawn on calcification periods of *Cytheridella* and its geographical distribution. Offsets between *Cytheridella* δ^18^O and the estimated monthly expected equilibrium calcites vary throughout the year and coincide with the required vital offset of ~  + 1‰ during spring (April/May) and autumn (October) which indicates that *Cytheridella* calcifies seasonally in all investigated regions. This implies a synchronous life cycle of *Cytheridella* for the studied wide geographical range. Since the regions differ in climatic conditions (i.e., precipitation seasonality and amounts, temperature gradients) an environmental control on *Cytheridella*’s life cycle is implausible; instead, phylogenetic relationships offer a much more probable explanation.

## Supplementary Information

Below is the link to the electronic supplementary material.

Supplementary Fig 1. Piper diagram illustrating the major ion compositions of the investigated solutions grouped according to regions Florida (FL), Mexico (MX), Brazil (BR), Panama (PA), and Colombia (CO) (TIF 2724 kb)

## Data Availability

All data generated or analysed during this study are included in this published article [and its supplementary information files].
