# Supplementary figures and images for: Neotropical ostracode oxygen and carbon isotope signatures: implications for calcification conditions
Source: Biogeochemistry. 2022 Mar 29;159(1):103–38. doi: 10.1007/s10533-022-00917-9 (PMC9042974; doi:10.1007/s10533-022-00917-9)

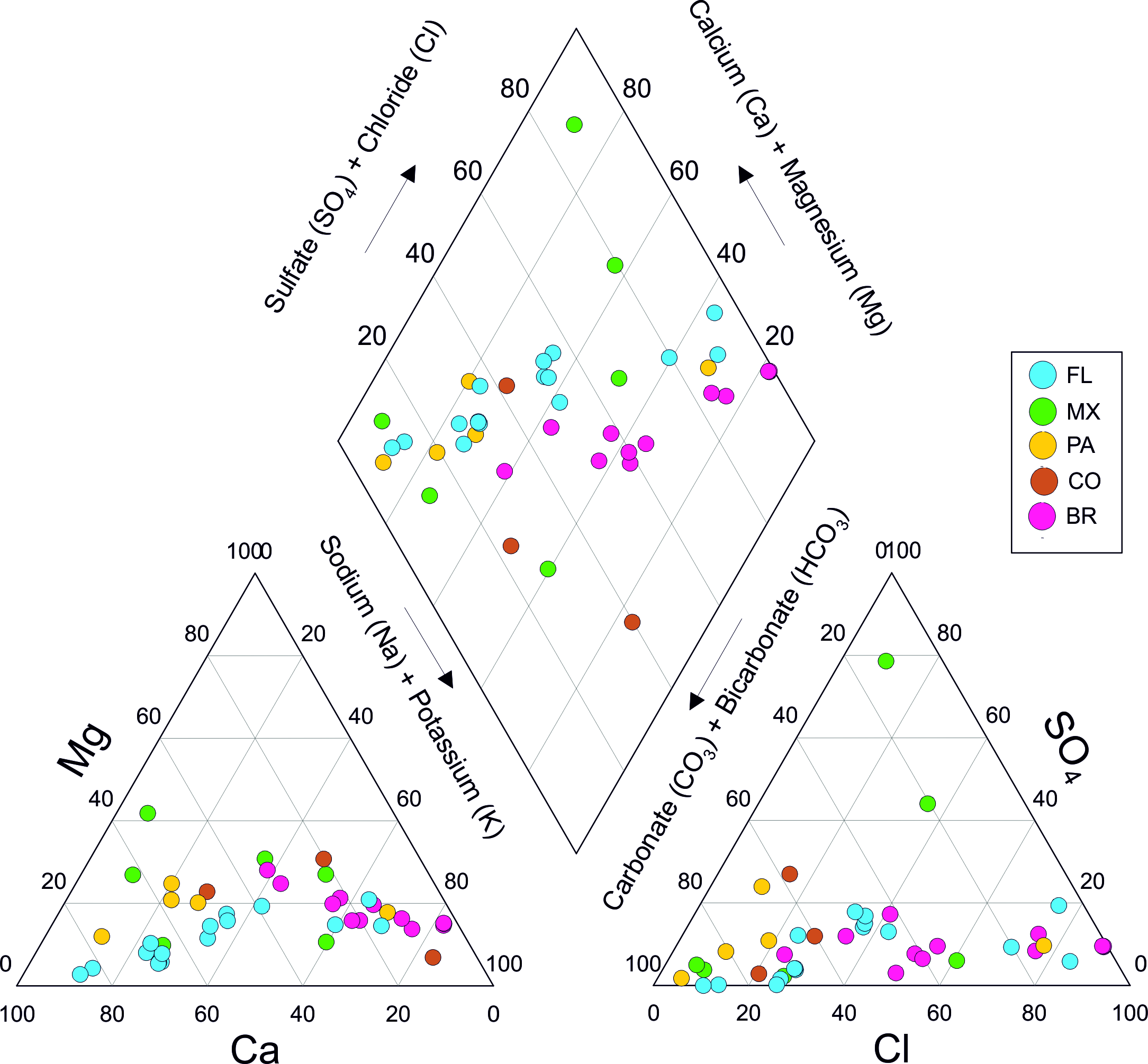

Supplement: Supplementary file 1 — Supplementary Fig 1. Piper diagram illustrating the major ion compositions of the investigated solutions grouped according to regions Florida (FL), Mexico (MX), Brazil (BR), Panama (PA), and Colombia (CO) (TIF 2724 kb) [file 10533_2022_917_MOESM1_ESM.tif]
